# Sprouty2 Regulates Endocytosis and Degradation of Fibroblast Growth Factor Receptor 1 in Glioblastoma Cells

**DOI:** 10.3390/cells13231967

**Published:** 2024-11-28

**Authors:** Barbara Hausott, Lena Pircher, Michaela Kind, Jong-Whi Park, Peter Claus, Petra Obexer, Lars Klimaschewski

**Affiliations:** 1 Institute of Neuroanatomy, Medical University of Innsbruck, 6020 Innsbruck, Austria; lena.pircher@yahoo.de (L.P.); michaela.kind@i-med.ac.at (M.K.); jpark@gachon.ac.kr (J.-W.P.); lars.klimaschewski@i-med.ac.at (L.K.); 2 Institute of Functional and Applied Anatomy, Hannover Medical School, 30625 Hannover, Germany; claus.peter@mh-hannover.de; 3 Center for Systems Neuroscience, 30625 Hannover, Germany; 4 Department of Pediatrics II, Medical University of Innsbruck, 6020 Innsbruck, Austria; petra.obexer@i-med.ac.at

**Keywords:** caveolin-1, c-casitas b-lineage lymphoma, clathrin, extracellular signal-regulated kinase, phospholipase Cγ1, ubiquitin

## Abstract

The Sprouty (SPRY) proteins are evolutionary conserved modulators of receptor tyrosine kinase (RTK) signaling. SPRY2 inhibits fibroblast growth factor (FGF) signaling, whereas it enhances epidermal growth factor (EGF) signaling through inhibition of EGF receptor (EGFR) endocytosis, ubiquitination, and degradation. In this study, we analyzed the effects of SPRY2 on endocytosis and degradation of FGF receptor 1 (FGFR1) using two human glioblastoma (GBM) cell lines with different endogenous SPRY2 levels. SPRY2 overexpression (SPRY2-OE) inhibited clathrin- and caveolae-mediated endocytosis of FGFR1, reduced the number of caveolin-1 vesicles and the uptake of transferrin. Furthermore, FGFR1 protein was decreased by SPRY2-OE, whereas EGFR protein was increased. SPRY2-OE enhanced FGFR1 degradation by increased c-casitas b-lineage lymphoma (c-CBL)-mediated ubiquitination, but it diminished binding of phospholipase Cγ1 (PLCγ1) to FGFR1. Consequently, SPRY2-OE inhibited FGF2-induced activation of PLCγ1, whereas it enhanced EGF-induced PLCγ1 activation. Despite the reduction of FGFR1 protein and the inhibition of FGF signaling, SPRY2-OE increased cell viability, and knockdown of SPRY2 enhanced the sensitivity to cisplatin. These results demonstrate that the inhibitory effect of SPRY2-OE on FGF signaling is at least in part due to the reduction in FGFR1 levels and the decreased binding of PLCγ1 to the receptor.

## 1. Introduction

Receptor tyrosine kinases (RTKs) are transmembrane receptors that transduce extracellular signals to the interior of the cell. They are of special interest due to their important roles in many diseases including cancer. Several mechanisms of RTK dysregulation were identified in tumors, including overexpression, oncogenic fusion, or activating mutations [1]. Amplifications and/or mutations of epidermal growth factor receptor (EGFR) occur in 57% of glioblastoma (GBM) patients [2]. By contrast, fibroblast growth factor receptor (FGFR) mutations and amplifications are less frequent in GBM [3].

Oncogenic fusions between FGFRs and the transforming acidic coiled-coil (TACC) occur in about 3% of GBM patients [4]. Although FGFR mutations are relatively rare in GBM compared to EGFR, FGFRs are involved in GBM progression and patient survival [3]. Among the four types of FGFRs (FGFR1–4), high expression of FGFR1 but not of FGFR2–4 correlates with reduced survival of GBM patients, and FGFR1 expression is increased in GBM compared to normal brain tissue [5,6]. Basic FGF (bFGF, FGF2) is an important ligand of FGFR1 in GBM, and together, they increase self-renewal and maintenance of GBM cancer stem cells, thereby driving tumor growth [7]. FGF2/FGFR1 induces resistance to radiotherapy in GBM, and inhibition of FGFR1 reduces radioresistance in GBM mouse xenografts, although tumor growth is not reduced by FGFR1 silencing alone without irradiation [8,9,10].

Following ligand-induced FGFR activation, receptors undergo dimerization, which leads to the transphosphorylation of their intracellular kinase domains and to the activation of multiple intracellular signaling pathways. Major pathways of FGFR signaling are the rat sarcoma (RAS)/extracellular signal-regulated kinase (ERK) pathway via fibroblast growth factor receptor substrate 2 (FRS2), growth factor receptor-bound protein (GRB2) and son of sevenless (SOS); the phosphoinositide 3-kinase (PI3K)/Akt pathway through FRS2, GRB2, and GTPase-activating protein 1 (GAP1); and phospholipase C (PLC)-dependent signaling mechanisms [11]. Since these pathways are involved in cellular proliferation and survival, a regulatory system that tightly controls and balances RTK activation is required. Major mechanisms that control FGFR-dependent signaling are endocytosis, endocytic sorting, phosphorylation/dephosphorylation, and negative regulatory proteins such as Sprouty (SPRY) or Sprouty-related EVH1 domain (SPRED) proteins [12].

RTK activation is followed by internalization of the receptor and the ligand. Endocytosed receptors are then further transported into multivesicular bodies/lysosomes for degradation, resulting in attenuation of signaling. Alternatively, receptors are recycled back to the plasma membrane, providing sustained signaling [12]. FGFR1 is mainly internalized via clathrin-mediated endocytosis (CME) independently of the ligand concentration, but caveolin-mediated endocytosis has been observed as well [13,14,15,16,17]. Receptor dimerization and phosphorylation of serine 789 and tyrosine 766, the binding site for PLCγ1, are essential for FGFR1 endocytosis [18,19,20,21]. Intracellular sorting of internalized FGFRs to lysosomes or to recycling endosomes is regulated by receptor ubiquitination through the E3 ubiquitin ligases c-casitas b-lineage lymphoma (c-CBL) via FRS2/GRB2 or neural precursor cell-expressed developmentally downregulated protein 4 (NEDD4). Reduced ubiquitination of FGFR1 leads to enhanced recycling and decreased FGFR1 degradation [13,22,23,24]. By contrast, receptor ubiquitination or c-CBL activity are not required for FGFR1 endocytosis [13].

SPRY proteins were originally identified as antagonists of FGF and EGF signaling in *Drosophila*, controlling apical branching of the airways and eye development of the fruit fly [25,26,27]. Four SPRY homologs (SPRY1-4) were identified in mice and humans, with SPRY2 exhibiting the highest sequence homology to *Drosophila* SPRY (dSPRY), indicating its distinct evolutionary conservation [28,29,30,31]. SPRY2 reduces RAS/ERK signaling in response to FGF, nerve growth factor (NGF), brain-derived neurotrophic factor (BDNF), or glial cell-line-derived neurotrophic factor (GDNF) [32,33,34,35]. Through the interaction with GRB2 and rapidly accelerated fibrosarcoma (RAF), SPRY2 interferes with the ERK pathway upstream and downstream of RAS [29,36,37]. In addition to the RAS/ERK pathway, SPRY2 inhibits the activation of PI3K/Akt and PLCγ [38,39,40]. Although the major function of SPRY2 is the inhibition of RTK signaling, it acts as an oncogene in colon cancer and in GBM, while in most other tumors, it fulfills its canonical role as tumor suppressor [41,42]. Among the four SPRY isoforms, SPRY2 is upregulated in GBM patients, which correlates with reduced survival. Furthermore, SPRY2 promotes tumor growth of GBM mouse xenograft models [43].

In comparison to other growth factors, EGF-mediated ERK activation is differently controlled by SPRY2. Depending on the model system used, it has either no effect, an inhibitory, or a signal enhancing effect [43,44,45,46,47]. SPRY2 induces sustained EGF-induced ERK signaling by the attenuation of EGFR endocytosis, ubiquitination, and degradation through its binding to the RING finger domain of c-CBL [46,47,48]. The phosphorylation of tyrosine 55 at the N terminus of SPRY2 functions as a docking site for the SH2 domain of c-CBL, thereby competing with the activated EGFR for binding to c-CBL. Thus, SPRY2 is ubiquitinated instead of EGFR, which leads to sustained EGF signaling [46,47,48,49,50]. Similarly, SPRY2 increases FGF2-induced ERK activation by decreased ubiquitination of FGFR3 in COS-7 cells [51].

In this study, we used the two human GBM cell lines U251 and SF126 to study the effects of SPRY2 on endocytosis and degradation of FGFR1. SPRY2 overexpression (SPRY2-OE) inhibited clathrin- and caveolae-mediated endocytosis of FGF2/FGFR1 and reduced the uptake of transferrin in U251 cells, whereas SPRY2 downregulation (shSPRY2) increased endocytosis of FGFR1 and uptake of transferrin in SF126 cells. SPRY2-OE increased degradation of FGFR1 by enhanced c-CBL-mediated ubiquitination in U251 cells. In addition, SPRY2-OE reduced binding of PLCγ1 to FGFR1 and inhibited FGF2-induced activation of PLCγ1, whereas it reduced EGFR degradation and potentiated EGF-induced PLCγ1 signaling. Furthermore, SPRY2-OE increased the cell viability of U251 cells, and knockdown of SPRY2 enhanced the sensitivity of SF126 cells to cisplatin treatment.

## 2. Materials and Methods

### 2.1. Cell Culture and Transfection

The human GBM cell lines U251 and SF126 were authenticated by short tandem repeat analysis (Microsynth, Balgach, Switzerland) and cultured in Roswell Park Memorial Institute Medium (RPMI, Gibco, Vienna, Austria) with 5% fetal bovine serum (FBS, Gibco) and 1% antibiotic/antimycotic (Gibco) or in Dulbecco’s Modified Eagle’s Medium (DMEM, Sigma-Aldrich, Vienna, Austria) supplemented with 10% FBS, 2 mM L-glutamine (Gibco), and 1% antibiotic/antimycotic, respectively. As demonstrated in our previous studies, U251 cells reveal low endogenous SPRY2 levels, whereas SF126 cells have high endogenous SPRY2 levels. As previously described, U251 cells with stable SPRY2 overexpression (SPRY2-OE) were established by lentiviral expression. The mutant SPRY2^Y55F^, in which the binding site for c-CBL (tyrosine 55) is mutated to phenylalanine, was generated using site-directed mutagenesis, and stable overexpression of SPRY2^Y55F^ in U251 cells was established by lentiviral expression. For short hairpin (sh)RNA-mediated SPRY2 depletion, the oligonucleotide (shRNA target: GCAGGTACATGTCTTGTCT) was inserted into the pGLTR-puro plasmid for stable and conditional RNA interference. SF126 cells were transduced with lentiviral vectors for doxycycline-inducible SPRY2 shRNA (shSPRY2) expression [43,52]. Doxycycline (1 µg/mL; Sigma-Aldrich) was added to the cell culture medium three days before the start of the experiment to induce SPRY2 knockdown. All cells were cultured under standard conditions in a humidified atmosphere at 37 °C with 5% CO2. U251 and SF126 naive control cells, U251 cells with stable SPRY2-OE or SPRY2^Y55F^, and SF126 cells with shSPRY2 were transiently transfected with FGFR1 fused to enhanced green fluorescent protein (FGFR1-EGFP) or EGFR-EGFP using DharmaFect kb DNA transfection reagent (Dharmacon, Vienna, Austria). U251 control cells and U251 cells with stable SPRY2-OE were transfected with ON-TARGETplus non-targeting Control siRNA (siControl; D-001810-10-05) or ON-TARGETplus SMARTpool c-CBL siRNA (siCBL; L-HUMAN-XX-0005) from Dharmacon using the DharmaFect kb transfection reagent. Forty-eight hours after siRNA transfection, cells were transfected with the FGFR1-EGFP plasmid.

### 2.2. FGF2 Purification and Labeling

The 18 kDa isoform of His-tagged FGF2 was expressed in *Escherichia coli* BL21 (DE3-trx) after isopropyl β-D-1-thiogalactopyranoside (IPTG) induction and growth at 30 °C. Recombinant 18 kDa FGF2 was purified as previously described [53] with the following modification: After the first purification of the His-tagged protein on Ni-beads, FGF2 was eluted, and an additional purification step was performed on Heparin Sepharose. After high-salt elution with 1.5 M NaCl, the protein was dialyzed against phosphate-buffered saline (PBS) at 4 °C overnight. Subsequently, recombinant FGF2 was fluorescently labeled with cyanine 3 (Cy3) maleimide mono-reactive dye (Amersham, Darmstadt, Germany) according to the manufacturer’s protocol.

### 2.3. Immunostaining and Fluorescent Markers

Cells were treated for 30 min with Cy3-labeled FGF2 (FGF2-Cy3; 200 ng/mL) and heparan sulfate sodium salt (10 µg/mL; Sigma-Aldrich) 24 h after transfection with FGFR1-EGFP and incubated with transferrin Alexa Fluor 647 conjugate (transferrin-647; 1:200, Invitrogen, Vienna, Austria) for 15 min, washed, and fixed with 4% buffered paraformaldehyde (PFA, Sigma-Aldrich) for 15 min at room temperature (RT). For immunostaining, cells were treated with Cy3-labeled FGF2 (200 ng/mL) plus heparan sulfate sodium salt (10 µg/mL) for 30 min, fixed for 15 min at RT with 4% PFA, permeabilized with 0.5% Triton X-100 (Sigma-Aldrich) for 10 min, and blocked against unspecific binding with 10% normal goat serum (Sigma-Aldrich) for 1 h at RT. Primary antibodies (Table 1) against clathrin heavy chain (1:50) or caveolin-1 (1:400) were diluted in 0.3% bovine serum albumin (BSA; Sigma-Aldrich)/PBS and incubated overnight at 4 °C. After washing, the secondary antibody (goat anti-rabbit Alexa Fluor 647, Invitrogen) diluted 1:1000 in 0.3% BSA/PBS was incubated for 2 h at RT. Then, the cells were embedded with ProLong Diamond Antifade Mountant (Invitrogen).

### 2.4. Imaging and Image Analysis

Confocal laser scanning microscopy of whole cells was performed to ensure that not only a selective section of the cells was analyzed. The TCS SP5 microscope (Leica, Vienna, Austria) was used with a 64× oil objective (N.A. = 1.4) and a scanning rate according to the Nyquist–Shannon sampling theorem. The 488 nm argon-ion laser was used to excite FGFR1-EGFP, the 561 nm DPSS laser for FGF2-Cy3, and the helium-neon (HeNe) 633 nm laser for 647 nm (clathrin, caveolin-1, and transferrin-647). Laser intensities were kept constant for the comparison of different experiments, and z-stacks with 0.13 µm slice spacing were acquired. Confocal images of single cells with medium overexpression levels of FGFR1-EGFP were converted from the LIF file format (version 3.7.2; Leica Application Suite X LAS X, Leica, Wetzlar, Germany) into the IMS file format of the Imaris software (version 9.7.1; Oxford Instruments, Wiesbaden, Germany). For image analysis, the region of interest (ROI) was defined by manual cropping of a single cell, and 15 to 20 cells per experiment were analyzed. The intensity threshold was set for each channel separately, and the vesicle analysis data were saved in the CSV file format. The number of vesicles per cell for each channel was counted using Excel software (version 2016; Microsoft Corporation, Vienna, Austria). Vesicles larger than one µm^3^ were excluded from the analysis. Colocalization analysis was performed using Imaris and Excel software. The ‘ImarisColoc’ tool was used to define a threshold for the colocalization of each set of experiments. Imaris records the intensity of all three channels simultaneously, and the defined threshold was used for each channel using Excel software. The presented images are original, uncropped images that were deconvolved using the ‘Confocal_Normal’ template in Huygens Professional software (version 22.04; Scientific Volume Imaging SVI, Hilversum, The Netherlands).

### 2.5. Western Blotting and Immunoprecipitation

For immunoprecipitation (IP), U251 control cells and U251 cells with SPRY2-OE were treated for 10 min with FGF2 (100 ng/mL) and heparan sulfate sodium salt (10 µg/mL) 24 h after transfection with FGFR1-EGFP. Cell lysates were prepared using IP lysis buffer (50 mM Tris/HCl pH = 7.5, 10% glycerol, 150 mM NaCl, 1% Nonidet P-40, 0.25% sodium deoxycholate, and 2 mM EDTA pH = 8) supplemented with 2 mM N-ethylmaleimide (Sigma-Aldrich), protease inhibitor cocktail (MedChemExpress, Vienna, Austria), and phosphatase inhibitor cocktail II and III (Sigma-Aldrich). Dynabeads M-280 sheep anti-rabbit IgG (Invitrogen) were conjugated with FGFR1 antibody (1:50) overnight at 4 °C. Protein lysates (500 µg) were incubated with anti-FGFR1-labeled beads for 1 h at 4 °C. Beads without antibody incubation were used as negative controls. After washing, the beads were boiled in loading buffer (LI-COR Biosciences, Bad Homburg, Germany). IP lysates and whole-cell lysates were separated by sodium dodecyl sulfate polyacrylamide gel electrophoresis (SDS-PAGE) and transferred to the Immobilon-FL-PVDF membrane (Millipore, Vienna, Austria). Naive U251 control cells, U251 cells with SPRY2-OE or SPRY2^Y55F^, naive SF126 control cells, and SF126 cells with shSPRY2 were treated for 30 or 120 min with FGF2 (100 ng/mL) and heparan sulfate sodium salt (10 µg/mL) 24 h after transfection with FGFR1-EGFP or for 30 or 120 min with EGF (100 ng/mL; Sigma-Aldrich) 24 h after transfection with EGFR-EGFP. For knockdown of c-CBL, U251 control cells and U251 cells with SPRY2-OE were transfected with siControl or siCBL. After 48 h, the cells were transfected with FGFR1-EGFP for another 24 h and then treated with FGF2 (100 ng/mL) and heparan sulfate sodium salt (10 µg/mL) for 30 min. U251 control cells, U251 cells with SPRY2-OE, SF126 control cells, and SF126 cells with shSPRY2 treated with FGF2 (100 ng/mL) and heparan sulfate sodium salt (10 µg/mL) for 30 min were used to analyze the endogenous FGFR1 levels. Cell lysates were prepared using RIPA buffer (50 mM Tris/HCl, 500 mM NaCl, 1% Nonidet P-40, 0.5% sodium deoxycholate, and 0.1% sodium dodecyl sulfate). The buffer was supplemented with protease inhibitor cocktail (MedChemExpress) and phosphatase inhibitor cocktail II and III (Sigma-Aldrich). Proteins (20 µg) were separated by SDS-PAGE and transferred to the Immobilon-FL-PVDF membrane (Millipore). Membranes were blocked with Intercept (PBS) blocking buffer (LI-COR Biosciences) and incubated with primary antibodies (Table 1) diluted in Intercept blocking buffer with 0.2% Tween 20: c-CBL, 1:1000; caveolin-1, 1:1000; CD44, 1:1000; clathrin heavy chain, 1:1000; EGFR, 1:1000; ERK1/2, 1:1000; pERK1/2, 1:1000; FGFR1, 1:1000; GAPDH Mouse, 1:1000; GAPDH Rabbit, 1:1000; NEDD4, 1:000; PLCγ1, 1:1000; pPLCγ1, 1:1000; SPRY2, 1:500; SOX2, 1:1000; α-tubulin, 1:1000; β-tubulin, 1:1000; ubiquitin, 1:1000. Primary antibodies were incubated overnight at 4 °C. Secondary fluorescently labeled antibodies (IRDye 680RD goat anti-mouse and IRDye 800CW goat anti-rabbit, 1:20,000; LI-COR Biosciences) were incubated for 1 h at RT and detected by the Odyssey FC Imaging System (LI-COR Biosciences). The band intensities were quantified after background subtraction using the Image Studio Lite Software (version 5.2; LI-COR Biosciences).

### 2.6. Quantitative Real-Time Polymerase Chain Reaction (qRT-PCR)

RNA was extracted using the RNeasy Mini Kit including DNase-digestion (Qiagen, Hilden, Germany) according to the manufacturer’s protocol. Then, 1 µg of RNA template was used for cDNA synthesis with iScript cDNA Synthesis Kit (Bio-Rad, Vienna, Austria). Quantitative real-time polymerase chain reaction (qRT-PCR) was performed with the CFX Connect Real-Time PCR Detection System (Bio-Rad) in a final volume of 20 µL with the SsoAdvanced Universal SYBR Green Supermix (Bio-Rad) and QuantiTect Primer Assays (Qiagen) for FGFR1 (Hs_FGFR1_vb.1_SG, QT02407622), EGFR (Hs_EGFR_vc.1_SG, QT00999957), and HPRT1 (Hs_HPRT_1_SG, QT00059066). The qRT-PCR reactions were performed in 40 cycles with 15 s at 95 °C and 30 s at 60 °C.

### 2.7. Cell Viability Assay

Naive U251 control cells, U251 cells with SPRY2-OE, naive SF126 control cells, and SF126 cells with shSPRY2 were plated in triplicates in 96-well plates. After 24 h, cells were transfected with FGFR1-EGFP, and 24 h after transfection, cells were treated with FGF2 (100 ng/mL) plus heparan sulfate sodium salt (10 µg/mL) and 10, 15, or 30 µM cisplatin in serum-reduced medium (RPMI with 0.5% FBS for U251 cells; DMEM with 1% FBS for SF126 cells). Twenty-four hours after treatment, the number of viable cells was determined using EZ4U/MTT assay according to the manufacturer’s instructions (Biomedica, Vienna, Austria).

### 2.8. Statistics

Data presented as bar graphs are mean values ± standard error of the mean (SEM) for the pooling of mean values or standard deviation (SD) for the pooling of single values of at least three independent experiments. The analysis employed paired *t*-test for the comparison of two groups. In cases involving more than two groups and independent variables, two-way analysis of variance (ANOVA) followed by Tukey’s post hoc test was performed. All statistical analyses were carried out using GraphPad PRISM software (version 8; GraphPad Software, Inc., Boston, MA, USA). Differences with a *p* < 0.05 were considered statistically significant (* *p* < 0.05, ** *p* < 0.01, *** *p* < 0.001, or **** *p* < 0.0001).

## 3. Results

### 3.1. SPRY2-OE Reduces but shSPRY2 Increases Endocytosis of FGFR1 and FGF2 and Their Colocalization

**Figure 1:**
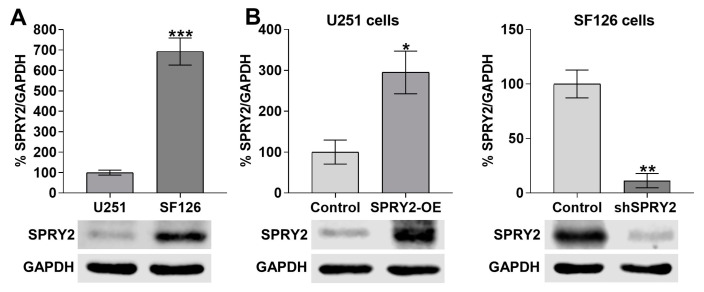
Western blot analyses of endogenous SPRY2 protein in U251 and SF126 cells and the effects of SPRY2 overexpression (SPRY2-OE) and SPRY2 short hairpin RNA (shSPRY2). (**A**) Endogenous SPRY2 protein is much lower in U251 cells than in SF126 cells. N = 4, mean ± SD. *** *p* < 0.001. (**B**) SPRY2-OE increased SPRY2 protein levels in U251 cells, whereas shSPRY2 reduced SPRY2 protein in SF126 cells. N = 4, mean ± SD. * *p* < 0.05, ** *p* < 0.01.

**Figure 2:**
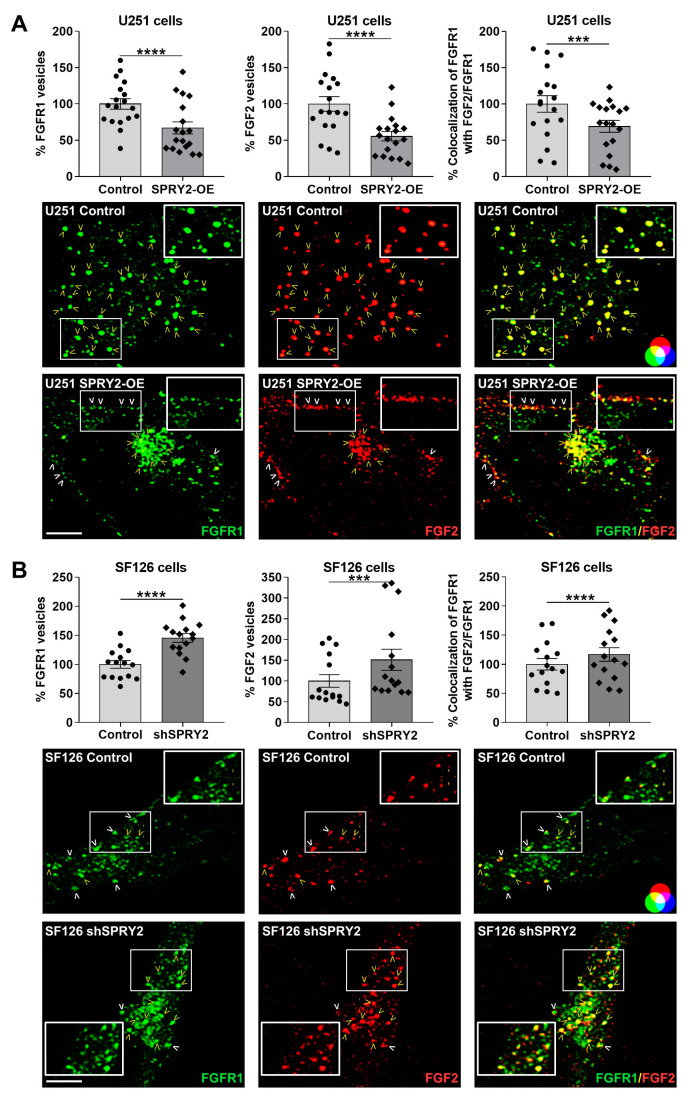
SPRY2 reduces the number of FGFR1 and FGF2 vesicles and their colocalization in U251 and SF126 cells. (**A**) U251 control cells with low endogenous SPRY2 level and U251 cells with SPRY2-OE were transfected with FGFR1 fused to enhanced green fluorescent protein (FGFR1-EGFP) and treated with cyanine 3-labeled FGF2 (FGF2-Cy3) for 30 min. Whole-cell analysis of confocal images revealed a reduction in FGFR1 (green) and FGF2 (red) vesicles per cell and their reduced colocalization (yellow) in response to SPRY2-OE. N = 18 experiments, mean ± SEM. *** *p* < 0.001, **** *p* < 0.0001. (**B**) SF126 control cells with high endogenous SPRY2 content and SF126 cells with shSPRY2 were transfected with FGFR1-EGFP and treated with FGF2-Cy3 for 30 min. Whole-cell analysis of confocal images revealed an increase in FGFR1 (green) and FGF2 (red) vesicles per cell as well as their enhanced colocalization (yellow) in response to shSPRY2. N = 15 experiments, mean ± SEM. *** *p* < 0.001, **** *p* < 0.0001. White, bold arrowheads indicate cell surface localization of FGFR1 and FGF2. Yellow arrowheads mark internalized FGFR1 vesicles colocalizing with FGF2. Scale bar = 4 µm.

To investigate the effects of SPRY2 on FGFR1 endocytosis and degradation, we used the two human GBM cell lines U251 and SF126, with low and high endogenous SPRY2 protein content, as described before [43,52]. Due to the genetic heterogeneity of GBM cell lines [54], SPRY2 was overexpressed (SPRY2-OE) in U251 cells with low endogenous SPRY2 levels and downregulated (shSPRY2) in SF126 cells with high endogenous SPRY2 protein (Figure 1A,B). Both cell lines exhibiting low endogenous FGFR1 levels (Figure S1) were transiently transfected with FGFR1-EGFP and treated with FGF2-Cy3. Whole-cell analysis of confocal images revealed that FGFR1 and FGF2 vesicles were reduced in U251 cells in response to SPRY2-OE, and more FGFR1 and FGF2 vesicles were retained at the cell surface. The colocalization rate between FGFR1 and FGF2, which was normalized to the reduced FGFR1 vesicles, was also decreased following SPRY2-OE (Figure 2A). In SF126 cells with high endogenous SPRY2 content, shSPRY2 enhanced the number of FGFR1 and FGF2 vesicles, and their colocalization that was normalized to the increased FGFR1 vesicles. In comparison, more FGFR1 and FGF2 vesicles remained at the cell surface of naive SF126 control cells with high endogenous SPRY2 content (Figure 2B).

### 3.2. SPRY2-OE Inhibits but shSPRY2 Enhances Clathrin- and Caveolae-Mediated Endocytosis of FGFR1 and FGF2

**Figure 3:**
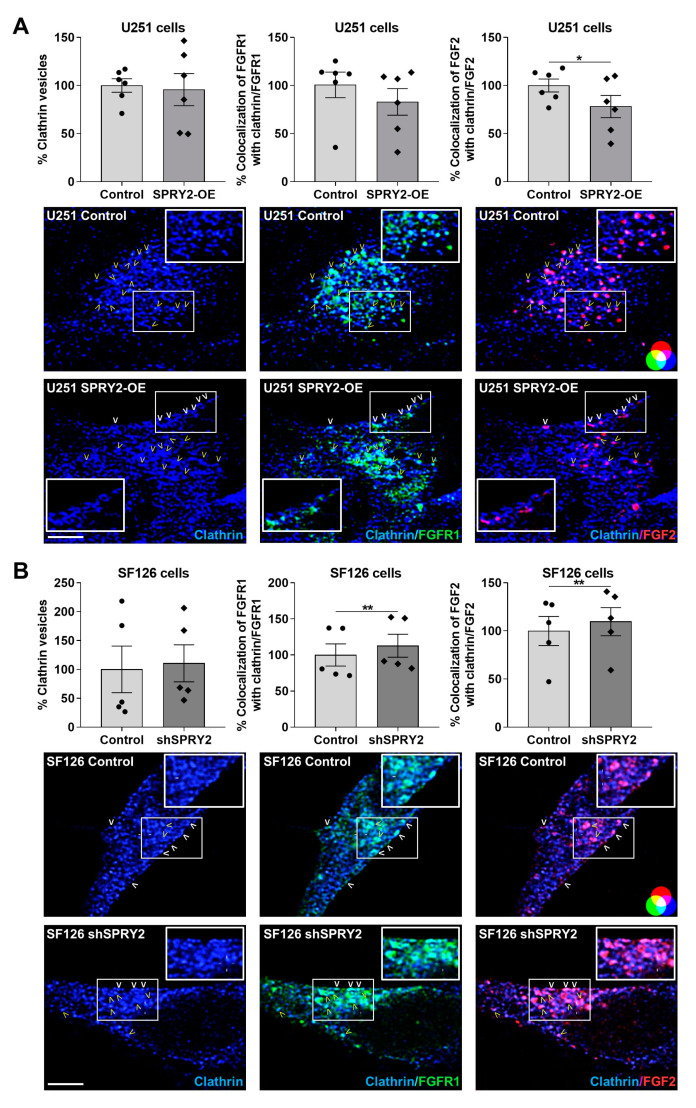
SPRY2 inhibits colocalization of clathrin with FGFR1 and FGF2 in U251 and SF126 cells. (**A**) U251 control cells with low endogenous SPRY2 level and U251 cells with SPRY2-OE were transfected with FGFR1-EGFP, treated with FGF2-Cy3 for 30 min, and immunostained against clathrin. Whole-cell analysis of confocal images revealed no change in the number of clathrin vesicles per cell (blue) after SPRY2-OE. The colocalization of clathrin (blue) with FGFR1 (green; colocalization with clathrin = turquoise) and FGF2 (red; colocalization with clathrin = magenta) was reduced in response to SPRY2-OE. N = 6 experiments, mean ± SEM. * *p* < 0.05. (**B**) SF126 control cells with high endogenous SPRY2 content and SF126 cells with shSPRY2 were transfected with FGFR1-EGFP, treated with FGF2-Cy3 for 30 min, and immunostained against clathrin. The number of clathrin vesicles per cell (blue) was not altered with shSPRY2. The colocalization of clathrin (blue) with FGFR1 (green; colocalization with clathrin = turquoise) and FGF2 (red; colocalization with clathrin = magenta) was enhanced with shSPRY2. N = 5 experiments, mean ± SEM. ** *p* < 0.01. White, bold arrowheads indicate cell surface localization of FGFR1 and FGF2. Yellow arrowheads mark internalized FGFR1 and FGF2 vesicles colocalizing with clathrin. Scale bar = 4 µm.

**Figure 4:**
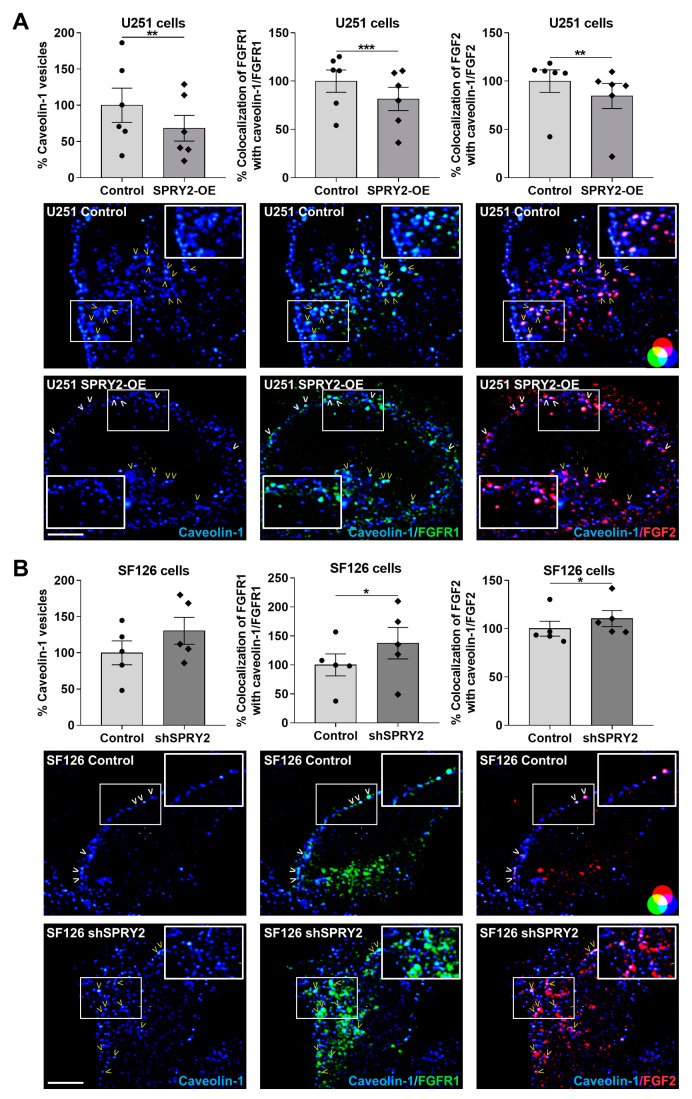
SPRY2 inhibits colocalization of caveolin-1 with FGFR1 and FGF2 in U251 and SF126 cells and reduces caveolin-1 vesicles. (**A**) U251 control cells with low endogenous SPRY2 level and U251 cells with SPRY2-OE were transfected with FGFR1-EGFP, treated with FGF2-Cy3 for 30 min, and immunostained against caveolin-1. Whole-cell analysis of confocal images revealed a reduction in the number of caveolin-1 vesicles per cell (blue) after SPRY2-OE. The colocalization of caveolin-1 (blue) with FGFR1 (green; colocalization with caveolin-1 = turquoise) and FGF2 (red; colocalization with caveolin-1 = magenta) was reduced in response to SPRY2-OE. N = 6 experiments, mean ± SEM. ** *p* < 0.01, *** *p* < 0.001. (**B**) SF126 control cells with high endogenous SPRY2 content and SF126 cells with shSPRY2 were transfected with FGFR1-EGFP, treated with FGF2-Cy3 for 30 min, and immunostained against caveolin-1. The number of caveolin-1 vesicles per cell (blue) was slightly but not significantly enhanced with shSPRY2. The colocalization of caveolin-1 (blue) with FGFR1 (green; colocalization with caveolin-1 = turquoise) and FGF2 (red; colocalization with caveolin-1 = magenta) was enhanced with shSPRY2. N = 5 experiments, mean ± SEM. * *p* < 0.05. White, bold arrowheads indicate cell surface localization of FGFR1 and FGF2. Yellow arrowheads mark internalized FGFR1 and FGF2 vesicles colocalizing with caveolin-1. Scale bar = 4 µm.

**Figure 5:**
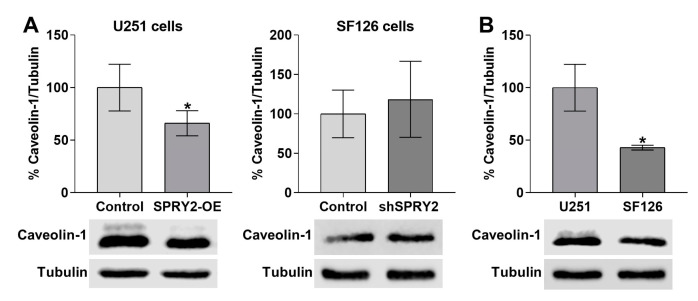
SPRY2 reduces caveolin-1 protein in U251 and SF126 cells. (**A**) SPRY2-OE reduced caveolin-1 protein levels in U251 cells and shSPRY2 slightly enhanced caveolin-1 protein in SF126 cells. N = 4 experiments, mean ± SD. * *p* < 0.05. (**B**) U251 cells with low endogenous SPRY2 revealed higher caveolin-1 protein levels than SF126 cells with high endogenous SPRY2. N = 4 experiments, mean ± SD. * *p* < 0.05.

In order to explore if these effects of SPRY2 on FGFR1 vesicles were related to FGFR1 endocytosis, we used clathrin heavy chain and caveolin-1 as two specific markers for endocytosis and analyzed colocalization of FGFR1/FGF2 with clathrin and caveolin-1 normalized to the number of FGFR1 and FGF2 vesicles, respectively. In U251 cells with low endogenous SPRY2 levels, SPRY2-OE reduced the colocalization of FGFR1 with clathrin non-significantly, whereas the colocalization of FGF2 with clathrin was significantly decreased. The number of clathrin vesicles was not changed by SPRY2 (Figure 3A). In SF126 cells with high endogenous SPRY2 content, shSPRY2 increased the colocalization of FGFR1 and FGF2 with clathrin, and the number of clathrin vesicles was again not changed (Figure 3B). Furthermore, SPRY2-OE reduced the number of caveolin-1 vesicles and the colocalization of FGFR1 and FGF2 with caveolin-1 in U251 cells (Figure 4A). ShSPRY2 enhanced the colocalization of FGFR1 and FGF2 with caveolin-1 in SF126 cells and increased the number of caveolin-1 vesicles slightly but not significantly (Figure 4B). Western blot analyses confirmed the reduction of caveolin-1 protein by SPRY2-OE in U251 cells and the slight but not significant increase in caveolin-1 protein by shSPRY2 in SF126 cells (Figure 5A). Furthermore, naive U251 cells with low endogenous SPRY2 content had higher caveolin-1 protein levels than naive SF126 cells with high endogenous SPRY2 protein (Figure 5B).

### 3.3. SPRY2-OE Decreases but shSPRY2 Enhances Transferrin Uptake and Colocalization of FGFR1 and FGF2 with Transferrin

**Figure 6:**
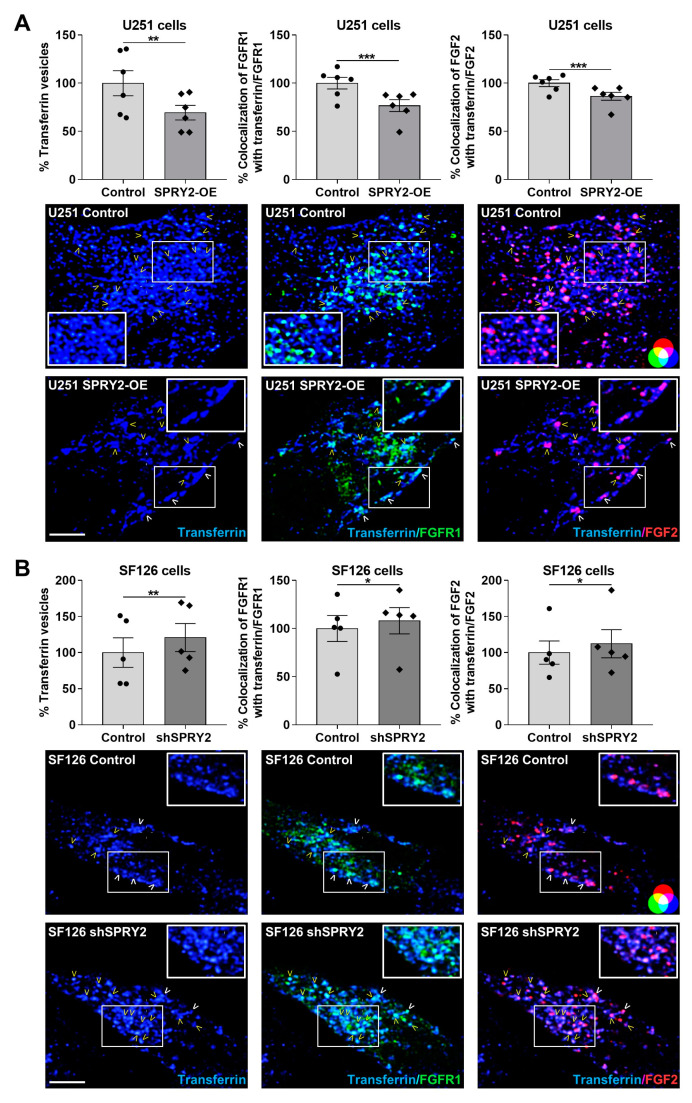
SPRY2 decreases transferrin-647 uptake and colocalization of transferrin with FGFR1 and FGF2 in U251 and SF126 cells. (**A**) U251 control cells with low endogenous SPRY2 level and U251 cells with SPRY2-OE were transfected with FGFR1-EGFP and treated with FGF2-Cy3 for 30 min and with transferrin-647 for 15 min. Whole-cell analysis of confocal images revealed a reduction in the uptake of transferrin vesicles per cell (blue) after SPRY2-OE. The colocalization of transferrin (blue) with FGFR1 (green; colocalization with transferrin = turquoise) and FGF2 (red; colocalization with transferrin = magenta) was also reduced in response to SPRY2-OE. N = 6 experiments, mean ± SEM. ** *p* < 0.01, *** *p* < 0.001. (**B**) SF126 control cells with high endogenous SPRY2 content and SF126 cells with shSPRY2 were transfected with FGFR1-EGFP and treated with FGF2-Cy3 for 30 min and with transferrin-647 for 15 min. The uptake of transferrin vesicles per cell (blue) was increased with shSPRY2, and the colocalization of transferrin (blue) with FGFR1 (green; colocalization with transferrin = turquoise) and FGF2 (red; colocalization with transferrin = magenta) was enhanced with shSPRY2. N = 5 experiments, mean ± SEM. * *p* < 0.05, ** *p* < 0.01. White, bold arrowheads indicate cell surface localization of FGFR1 and FGF2. Yellow arrowheads mark FGFR1 and FGF2 vesicles colocalizing with transferrin. Scale bar = 4 µm.

To further confirm the inhibitory effect of SPRY2 on endocytosis, fluorescently labeled transferrin-647 was added to the cells. Transferrin binds to the transferrin receptor, a transmembrane glycoprotein, and enters the cell through CME [55]. SPRY2-OE reduced the uptake of transferrin into U251 cells and the colocalization of transferrin with FGFR1 and FGF2 (Figure 6A). In SF126 cells, the number of transferrin vesicles was increased with shSPRY2, and the colocalization of transferrin with FGFR1 and FGF2 was enhanced in response to SPRY2 knockdown (Figure 6B). These data confirm the inhibitory role of SPRY2 on endocytosis.

### 3.4. SPRY2-OE Reduces FGFR1 Protein but shSPRY2 Increases FGFR1 Protein, Whereas SPRY2-OE Increases and shSPRY2 Reduces EGFR Protein

**Figure 7:**
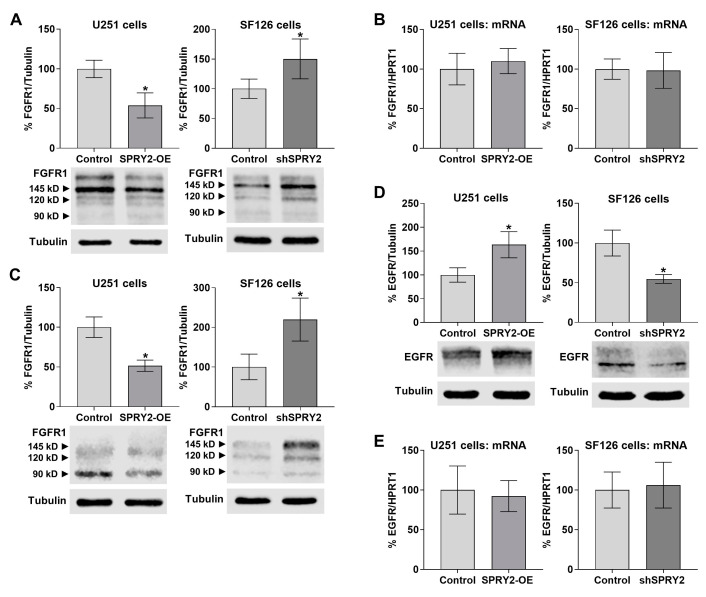
SPRY2 reduces FGFR1 and increases EGFR protein but has no effect on FGFR1 and EGFR mRNA levels. (**A**) Western blot analyses of U251 and SF126 cells overexpressing FGFR1-EGFP and treated with FGF2 for 30 min revealed reduced FGFR1 protein after SPRY2-OE in U251 cells and enhanced FGFR1 protein in SF126 cells with shSPRY2. N = 4 experiments, mean ± SD. * *p* < 0.05. (**B**) qRT-PCR did not reveal changes in FGFR1 mRNA content in response to SPRY2-OE in U251 cells or shSPRY2 in SF126 cells overexpressing FGFR1-EGFP and treated with FGF2 for 30 min. N = 3 experiments, mean ± SEM. (**C**) Western blot analyses of endogenous FGFR1 in U251 and SF126 cells that were not transfected with FGFR1-EGFP but treated with FGF2 for 30 min also revealed reduced FGFR1 protein after SPRY2-OE in U251 cells and enhanced FGFR1 protein in SF126 cells with shSPRY2. N = 4 experiments, mean ± SD. * *p* < 0.05. (**D**) Western blot analyses of U251 cells overexpressing EGFR-EGFP and treated with EGF for 30 min revealed increased EGFR protein after SPRY2-OE, whereas SF126 cells with shSPRY2 exhibited reduced EGFR protein. N = 3 experiments, mean ± SD. * *p* < 0.05. (**E**) qRT-PCR did not reveal changes in EGFR mRNA content in response to SPRY2-OE in U251 cells or shSPRY2 in SF126 cells overexpressing EGFR-EGFP and treated with EGF for 30 min. N = 3 experiments, mean ± SEM.

Western blot analyses confirmed reduced FGFR1 protein in U251 cells with SPRY2-OE, whereas FGFR1 protein was increased in SF126 cells by shSPRY2 in cells transfected with FGFR1-EGFP (Figure 7A). By contrast, FGFR1 mRNA levels were not changed, indicating that there is no effect of SPRY2 on FGFR1 transcription (Figure 7B). Although the Western blot signal of the endogenous FGFR1 was low and difficult to detect in both cell lines, the endogenous FGFR1 was also reduced in U251 cells with SPRY2-OE and enhanced in SF126 cells by shSPRY2, confirming the effects of SPRY2 on FGFR1 levels (Figure 7C).

Since SPRY2-OE increases EGFR protein in other cell lines [46,47], we compared the effects of SPRY2 on FGFR1 and EGFR levels in the same GBM cell lines. In contrast to FGFR1, U251 cells with SPRY2-OE revealed enhanced EGFR protein levels, whereas SF126 cells with shSPRY2 exhibited decreased EGFR protein in cells transfected with EGFR-EGFP (Figure 7D). EGFR mRNA levels were again not changed, confirming that there is no effect of SPRY2 on EGFR transcription (Figure 7E). These results confirm the different effect of SPRY2 on FGFR1 and EGFR protein levels in GBM cell lines.

### 3.5. SPRY2-OE Increases FGFR1 Degradation by Enhanced c-CBL-Mediated Ubiquitination and Reduces Binding of PLCγ1 to FGFR1

**Figure 8:**
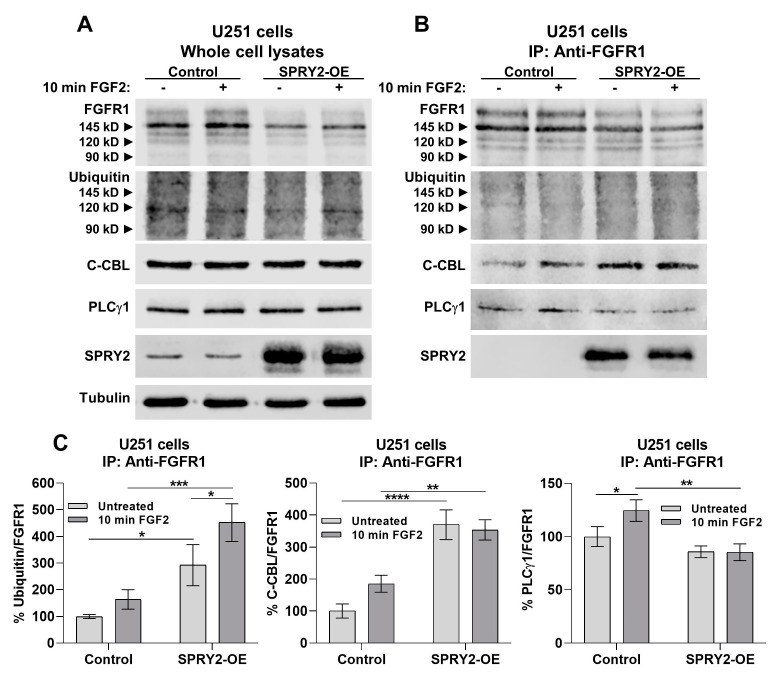
SPRY2-OE increases FGFR1 degradation by enhanced c-CBL-mediated ubiquitination and reduces binding of PLCγ1 to FGFR1 in U251 cells. (**A**) Whole-cell lysates of U251 control cells and U251 cells with SPRY2-OE transfected with FGFR1-EGFP and treated with FGF2 for 10 min. U251 cells with SPRY2-OE revealed reduced FGFR1 protein but no change in ubiquitin, c-CBL, or PLCγ1. (**B**) Anti-FGFR1 immunoprecipitates (IP) revealed reduced FGFR1 protein but enhanced ubiquitination and c-CBL after SPRY2-OE. PLCγ1 was reduced in anti-FGFR1 immunoprecipitates with SPRY2-OE, and the overexpressed but not the endogenous SPRY2 was detected in anti-FGFR1 immunoprecipitates. (**C**) Quantification of anti-FGFR1 immunoprecipitates (IP) confirmed the increase of ubiquitin and c-CBL but the reduction of PLCγ1 in response to SPRY2-OE. N = 3 experiments, mean ± SD. * *p* < 0.05, ** *p* < 0.01, *** *p* < 0.001, **** *p* < 0.0001.

Since SPRY2-OE reduces EGFR ubiquitination in COS-1 and CHO cells [46,47], we further analyzed the ubiquitination of FGFR1 by its E3 ubiquitin ligases performing anti-FGFR1 immunoprecipitation in U251 cells. In response to SPRY2-OE, the FGFR1 levels were again reduced in untreated whole-cell lysates to 78.3% (±6.9) and to 79.9% (±5.9) after 10 min FGF2 treatment. This reduction of FGFR1 protein in response to SPRY2-OE was also detected in untreated anti-FGFR1 immunoprecipitates to 82.9% (±3.5) and to 83.4% (±3.9) with FGF2 treatment (Figure 8A,B). FGFR1 was not detected in lysates incubated with plain beads without antibody incubation (Figure S2A). Although FGFR1 was reduced in anti-FGFR1 immunoprecipitates in response to SPRY2-OE, the ubiquitination of FGFR1 was enhanced with and without FGF2 treatment (Figure 8B,C). Furthermore, the E3 ubiquitin ligase c-CBL was increased in anti-FGFR1 immunoprecipitates in response to SPRY2-OE with and without FGF2 treatment compared to naive U251 control cells (Figure 8B,C). The E3 ubiquitin ligase NEDD4 was not detected in anti-FGFR1 immunoprecipitates, although it was present in whole-cell lysates of U251 cells (Figure S2B). In addition, PLCγ1 was significantly reduced in anti-FGFR1 immunoprecipitates, and the overexpressed but not the endogenous SPRY2 was detected in anti-FGFR1 immunoprecipitates (Figure 8B,C).

### 3.6. Knockdown of c-CBL Increases FGFR1 Protein in U251 Cells with SPRY2-OE, but Mutant SPRY2^Y55F^ Reduces FGFR1 Protein

**Figure 9:**
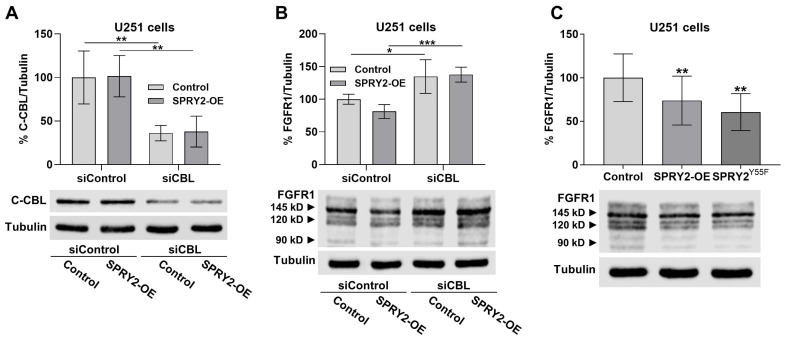
SiRNA-induced knockdown of c-CBL increases FGFR1 protein in U251 cells with SPRY2-OE but the mutant SPRY2^Y55F^, which does not bind c-CBL, reduces FGFR1 protein. (**A**) Western blot analyses of U251 cells transfected with scrambled control siRNA (siControl) or siRNA against c-CBL (siCBL) overexpressing FGFR1-EGFP and treated with FGF2 for 30 min revealed reduced c-CBL with siCBL compared to siControl in U251 control cells and in U251 cells with SPRY2-OE. N = 4 experiments, mean ± SD. ** *p* < 0.01. (**B**) U251 cells transfected with control siRNA (siControl) and siRNA against c-CBL (siCBL) overexpressing FGFR1-EGFP and treated with FGF2 for 30 min revealed increased FGFR1 protein in U251 control cells and in U251 cells with SPRY2-OE in response to siCBL. N = 5 experiments, mean ± SD. * *p* < 0.05, *** *p* < 0.001. (**C**) FGFR1 protein was reduced by SPRY2-OE and by overexpression of mutant SPRY2^Y55F^ in U251 cells overexpressing FGFR1-EGFP and treated with FGF2 for 30 min. N = 3 experiments, mean ± SD. ** *p* < 0.01.

To confirm that c-CBL is involved in the enhanced degradation of FGFR1 induced by SPRY2-OE, U251 control cells and U251 cells with SPRY2-OE were transfected with siRNA against c-CBL and FGFR1-EGFP. SiCBL reduced c-CBL protein in U251 control cells and U251 cells with SPRY2-OE after 72 h (Figure 9A). Although SPRY2-OE reduced FGFR1 protein, knockdown of c-CBL increased FGFR1 protein in U251 control cells and U251 cells with SPRY2-OE to the same extent (Figure 9B). The mutant SPRY2^Y55F^, which is unable to bind c-CBL [48,49,50], reduced overexpressed FGFR1 in the same manner as wild-type SPRY2-OE. These results demonstrate that although c-CBL is required for the increased degradation of FGFR1 by SPRY2-OE, binding of c-CBL to SPRY2 is not involved in this mechanism (Figure 9C).

### 3.7. SPRY2-OE Inhibits FGF2-Induced Activation of PLCγ1 and ERK, Whereas shSPRY2 Increases FGF2-Induced Activation of PLCγ1 but Not ERK

**Figure 10:**
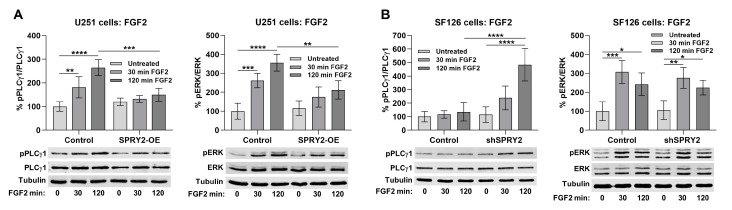
SPRY2-OE inhibits FGF2-induced activation of PLCγ1 and ERK in U251 cells, but shSPRY2 only increases FGF2-induced PLCγ1 activation in SF126 cells. (**A**) U251 control cells with low endogenous SPRY2 level and U251 cells with SPRY2-OE transfected with FGFR1-EGFP and treated with FGF2 for 30 and 120 min. Activation of PLCγ1 and ERK was inhibited by SPRY2-OE. N = 4 experiments, mean ± SD. ** *p* < 0.01, *** *p* < 0.001, **** *p* < 0.0001. (**B**) Naive SF126 control cells with high endogenous SPRY2 content and SF126 cells with shSPRY2 transfected with FGFR1-EGFP and treated with FGF2 for 30 and 120 min. Activation of PLCγ1 but not of ERK was elevated by shSPRY2. N = 4 experiments, mean ± SD. * *p* < 0.05, ** *p* < 0.01, *** *p* < 0.001, **** *p* < 0.0001.

Since SPRY2-OE decreased binding of PLCγ1 to FGFR1 and reduced FGFR1 levels but increased EGFR protein, we compared the effects of SPRY2 on activation of PLCγ1 and ERK in response to FGF2 and EGF. SPRY2-OE reduced FGF2-induced phosphorylation of PLCγ1 and ERK 30 and 120 min after FGF2 treatment (Figure 10A). In SF126 cells, shSPRY2 enhanced FGF2-induced phosphorylation of PLCγ1, whereas in SF126 control cells, PLCγ1 activation was completely blocked. In comparison, ERK was activated in SF126 control cells, and this effect was not influenced by shSPRY2 (Figure 10B).

### 3.8. SPRY2-OE Enhances EGF-Induced Activation of PLCγ1, Whereas shSPRY2 Reduces EGF-Induced Activation of PLCγ1 and ERK

**Figure 11:**
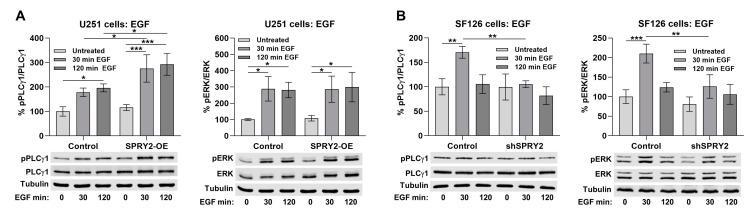
SPRY2-OE increases EGF-induced PLCγ1 signaling in U251 cells, and shSPRY2 inhibits EGF-induced PLCγ1 and ERK signaling in SF126 cells. (**A**) U251 control cells and U251 cells with SPRY2-OE transfected with EGFR-EGFP and treated with EGF for 30 and 120 min. Activation of PLCγ1 but not of ERK was increased by SPRY2-OE. N = 3 experiments, mean ± SD. * *p* < 0.05, *** *p* < 0.001. (**B**) Naive SF126 control cells and SF126 cells with shSPRY2 transfected with EGFR-EGFP and treated with EGF for 30 and 120 min. Activation of PLCγ1 and ERK was reduced by shSPRY2. N = 3 experiments, mean ± SD. ** *p* < 0.01, *** *p* < 0.001.

EGF-induced activation of PLCγ1 was enhanced after 30 and 120 min in response to SPRY2-OE in U251 cells, whereas activation of ERK was not changed by SPRY2-OE (Figure 11A). EGF-induced activation of PLCγ1 and ERK was inhibited by shSPRY2 in SF126 cells (Figure 11B). Together, these data demonstrate a more consistent effect of SPRY2 on PLCγ1 phosphorylation than on ERK activation in GBM cells, which correlates in both cell lines with its effects on FGFR1 and EGFR protein levels.

### 3.9. SPRY2-OE Increases Cell Viability of U251 Cells and shSPRY2 Increases the Sensitivity of SF126 Cells to Cisplatin Treatment

**Figure 12:**
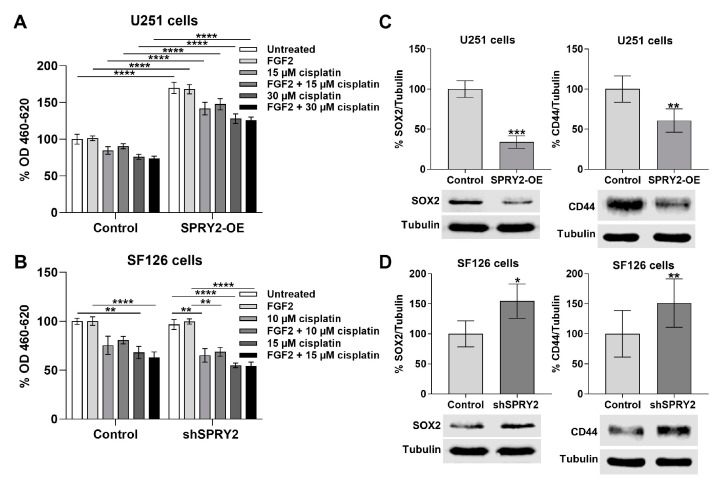
SPRY2-OE increases cell viability and reduces stemness markers in U251 cells, whereas shSPRY2 enhances cisplatin sensitivity and increases stemness markers in SF126 cells. (**A**) U251 control cells and U251 cells with SPRY2-OE were transfected with FGFR1-EGFP and treated with FGF2 and 15 and 30 µM cisplatin for 24 h. Cell viability of U251 cells was increased by SPRY2-OE in all groups. N = 4 experiments, mean ± SEM. **** *p* < 0.0001. (**B**) Naive SF126 control cells and SF126 cells with shSPRY2 were transfected with FGFR1-EGFP and treated with FGF2 and 10 and 15 µM cisplatin for 24 h. Cell viability of SF126 cells was not altered by shSPRY2, but cell viability was more strongly reduced by cisplatin treatment in cells with shSPRY2 than in control cells. N = 5 experiments, mean ± SEM. ** *p* < 0.01, **** *p* < 0.0001. (**C**) SPRY2-OE reduces SOX2 and CD44 in U251 cells overexpressing FGFR1-EGFP. N = 4 experiments, mean ± SD. ** *p* < 0.01, *** *p* < 0.001. (**D**) ShSPRY2 increases SOX2 and CD44 in SF126 cells overexpressing FGFR1-EGFP. N = 4 experiments, mean ± SD. * *p* < 0.05, ** *p* < 0.01.

The EZ4U/MTT cell viability assay demonstrated that SPRY2-OE increases proliferation of U251 cells overexpressing FGFR1-EGFP. This effect was not enhanced by FGF2 treatment, but 15 and 30 µM cisplatin reduced cell viability of U251 control cells and U251 cells with SPRY2-OE. However, cells viability of U251 cells with SPRY2-OE was still increased when treated with 15 and 30 µM cisplatin compared to U251 control cells (Figure 12A). Cell viability of SF126 cells with FGFR1-EGFP overexpression was not changed by shSPRY2. Since SF126 cells were more sensitive to 15 µM cisplatin than U251 cells, we also used 10 µM cisplatin for SF126 cells. FGF2 treatment, again, had no effect, but the reduction of SF126 cells with cisplatin was more pronounced in SF126 cells with shSPRY2 than in control cells with high endogenous SPRY2 levels. Therefore, 10 µM cisplatin reduced the cell number of SF126 control cells by 25%, but with shSPRY2, the cell viability was reduced by 35%. With 15 µM cisplatin, the cell viability of SF126 control cells was reduced by 32% but in response to SPRY2 knockdown by 45%. Similar effects were observed after co-treatment with FGF2 plus cisplatin (Figure 12B).

Stemness of GBM is involved in the resistance to cancer treatment. Therefore, we determined the stemness markers SOX2 and CD44 in response to SPRY2 regulation. SPRY2-OE reduced SOX2 and CD44 in U251 cells, although cell viability was increased (Figure 12C). In SF126 cells, shSPRY2 increased SOX2 and CD44 despite enhanced chemosensitivity to cisplatin (Figure 12D).

## 4. Discussion

SPRY2 is an inhibitor of FGF signaling, but it enhances EGF signaling by inhibition of EGFR endocytosis, ubiquitination, and degradation [46,47]. Furthermore, SPRY2 interferes with the transport of activated EGFR from early to late endosomes [56]. In the current study, we demonstrate that SPRY2-OE inhibits endocytosis of FGFR1, but it increases degradation of FGFR1 by enhanced c-CBL-mediated ubiquitination in GBM cells. Furthermore, SPRY2-OE reduces binding of PLCγ1 to FGFR1 and inhibits FGF2-induced activation of PLCγ1. Despite the reduction of FGFR1 protein and the inhibition of FGF signaling, SPRY2-OE increases the cell viability of U251 cells, and shSPRY2 increases the sensitivity of SF126 cells to cisplatin treatment.

SPRY2-OE reduced endocytosis of FGFR1 together with FGF2 in U251 cells, whereas shSPRY2 increased endocytosis of FGFR1/FGF2 in SF126 cells. Endocytosis via clathrin and caveolin-1 was inhibited by SPRY2-OE, and both pathways are involved in internalization of FGFR1, with CME being the major endocytic mechanism of FGFR1 [13,14,15,17]. Uptake of transferrin, which enters the cell through CME [55], was also reduced in U251 cells with SPRY2-OE and increased in SF126 cells with shSPRY2. Transferrin is used in different studies to explore inhibition of CME [57,58,59], and the effect of SPRY2-OE on the reduction of transferrin uptake confirms the inhibitory role of SPRY2 on endocytosis, which is not only caused by the reduction of FGFR1 vesicles. Furthermore, SPRY2-OE reduced the number of caveolin-1 vesicles and caveolin-1 protein in U251 cells, and shSPRY2 slightly increased caveolin-1 in SF126 cells. SPRY2 interacts with caveolin-1 [32,45], and caveolin-1 is often increased in GBM compared to normal brain tissue [60]. High caveolin-1 expression is associated with shorter survival of GBM patients and enhanced tumor invasion. Knockdown of caveolin-1 reduces the ability of U251 cells to invade through the basement membrane, and U118 GBM cells with undetectable levels of caveolin-1 are unable to invade [61,62].

Ubiquitination of FGFR1 by c-CBL was increased in response to SPRY2-OE in U251 cells, but neither ubiquitination nor c-CBL are required for FGFR1 endocytosis, whereas receptor dimerization and kinase activation are crucial for its endocytosis [13,18,20]. Mutation of tyrosine 766, the binding site for PLCγ1 in activated FGFR1, significantly reduces receptor internalization and degradation [18,21,63]. SPRY2-OE inhibited FGFR1 endocytosis and binding of PLCγ1 to FGFR1, indicating that SPRY2 may interfere with the activation of tyrosine 766 that is required for FGFR1 endocytosis. Furthermore, the p90 ribosomal S6 kinase 2 (RSK2) regulates endocytosis of FGFR1 by its phosphorylation on serine 789, and RSK2 is activated by the RAS/ERK pathway downstream of FGFR1 [19]. Since SPRY2-OE inhibited activation of ERK in U251 cells, RSK2 is not very likely to be involved in the inhibition of FGFR1 endocytosis by SPRY2.

Western blot analysis confirmed the reduction of FGFR1 protein by SPRY2-OE in untreated U251 cells and after FGF2 treatment. FGF secretion from the cells may contribute to this effect in untreated cells. Enhanced ubiquitination by the ubiquitin ligase c-CBL but not NEDD4 increased FGFR1 degradation in response to SPRY2-OE. Knockdown of c-CBL increased FGFR1 protein in U251 cells with SPRY2-OE, thereby confirming the role of c-CBL in SPRY2-induced FGFR1 degradation. By contrast, FGFR1 protein was increased by shSPRY2 in SF126 cells, whereas EGFR protein was enhanced by SPRY2-OE in U251 cells and reduced by shSPRY2 in SF126 cells, confirming a different effect of SPRY2 on degradation of FGFR1 and EGFR. The inhibition of EGFR degradation by SPRY2 through reduced receptor ubiquitination was observed in earlier studies using different cell lines [46,47]. Through the binding to the RING finger domain of c-CBL, SPRY2 interferes with EGFR internalization [64]. Tyrosine 55 at the N terminus of SPRY2 is phosphorylated by EGF and functions as a docking site for the SH2 domain of c-CBL, which competes with the activated EGFR [46,48,49,50]. The mutant SPRY2^Y55F^, which is unable to interact with c-CBL, reduced FGFR1 protein in the same way in U251 cells as overexpression of wild-type SPRY2, indicating that binding of c-CBL to SPRY2 is not involved in this mechanism. In contrast to our results, SPRY2 had no effects on ubiquitination of FGFR1 in HEK293T cells but reduced ubiquitination of FGFR3 in COS-7 fibroblasts, and ubiquitination of FGFR3 is partially restored by mutant SPRY2^Y55F^ [48,51]. Our results demonstrate that in U251 GBM cells, SPRY2-OE strongly enhances c-CBL-mediated ubiquitination and degradation of FGFR1, which reduces FGFR1 protein, whereas it increases EGFR protein. The major difference between FGFR1 and EGFR ubiquitination is the adaptor protein FRS2, which binds to a constitutive complex of c-CBL and GRB2 after FGFR1 activation, competing with binding of SOS to GRB2 [23]. SPRY2 directly interacts with FRS2, GRB2, and c-CBL [29,37,64,65], and overexpressed SPRY2 was precipitated with FGFR1 in U251 cells.

SPRY2-OE increased FGFR1 degradation and reduced FGF2 signaling, whereas it reduced EGFR degradation and potentiated EGF signaling. The receptor level correlated even more with the effects of SPRY2 on the activation of PLCγ1, which binds directly to FGFR1 and EGFR [21,66], than with its effects on ERK activation. In U251 cells, SPRY2-OE reduced FGF2-induced activation of PLCγ1 and ERK, but only phosphorylation of PLCγ1 and not ERK was enhanced by EGF. In SF126 cells, shSPRY2 increased FGF2-induced PLCγ1 but not ERK signaling, whereas it reduced EGF-induced ERK and PLCγ1 activation. Accordingly, increased FGFR4 levels selectively enhance PLCγ1 but not ERK signaling [67]. Other studies observed similar correlations between the effects of SPRY2 on FGFR and EGFR degradation and signaling. SPRY2 inhibits degradation of FGFR3, thereby enhancing FGF2-induced ERK activation, and it inhibits EGFR degradation and enhances EGF-induced ERK activation. The simultaneous inhibition of FGF signaling and potentiation of EGF signaling by SPRY2 has also been observed in PC12 cells, and this correlates with the effects of SPRY2 on FGF- and EGF-induced PC12 cell differentiation [46,47,51].

Our results demonstrate that SPRY2-OE enhances c-CBL-mediated ubiquitination and degradation of FGFR1, thereby reducing FGF2-induced activation of PLCγ1 and of ERK in human GBM cells. Inhibition of ERK signaling is a promising target for many types of tumors, but in GBM, enhanced ERK signaling in response to SPRY2 knockdown blocks intracranial tumor growth through premature onset of DNA replication, increased DNA damage, and impaired proliferation [43,68]. Cell viability of U251 cells overexpressing FGFR1-EGFP was strongly increased by SPRY2-OE even after cisplatin treatment, which confirms our previous study that SPRY2-OE increases tumor growth of U251 xenografts in mice [43]. Although the cell number of SF126 cells overexpressing FGFR1-EGFP was not altered by shSPRY2, the cell viability was more strongly reduced by cisplatin in SF126 cells with SPRY2 knockdown. In accordance with our results, previous studies demonstrated that knockdown of SPRY2 enhances cell death induced by UV radiation and the DNA-damaging agent cisplatin in HRas oncogene-transformed fibroblasts [69]. Radioresistance is a major problem in the treatment of GBM [70,71], and stemness of GBM is involved in the resistance to cancer treatment [72,73]. However, SPRY2-OE reduced the stemness markers SOX2 and CD44, whereas shSPRY2 increased these two GBM stemness markers. Recent studies demonstrated that knockdown of SPRY2 enhances CD44 in cancer-associated fibroblasts, whereas knockdown of SPRY1 reduces stemness of patient-derived glioma stem cell spheres [74,75]. Therefore, further studies investigating the influence of different SPRY isoforms on the stemness of GBM using molecular techniques such as RNA sequencing are required.

## 5. Conclusions

Taken together, our results demonstrate that SPRY2-OE inhibits clathrin- and caveolin-1-mediated endocytosis of FGFR1 and reduces the amount of caveolin-1 as well as the uptake of transferrin into GBM cells. In contrast to EGFR, SPRY2-OE enhances degradation of FGFR1 through c-CBL-mediated ubiquitination of the receptor and reduces binding of PLCγ1 to FGFR1, thereby inhibiting FGF2-induced activation of PLCγ1. Despite the reduction of FGFR1 protein and the inhibition of FGF signaling, SPRY2-OE increases cell viability, and knockdown of SPRY2 enhances the sensitivity to cisplatin treatment. Therefore, the current study reveals different and novel mechanisms of SPRY2 on RTK regulation, signaling and cell viability in GBM cells.

## Figures and Tables

**Table 1:** Primary antibodies used for immunostaining and Western blot experiments.

Antibody	Company	Catalog Number
C-CBL (D4E10)	Cell signaling, Leiden, The Netherlands	8447
Caveolin-1 (D46G3) XP	Cell signaling	3267
CD44 (E7K2Y)	Cell signaling	37259
Clathrin heavy chain (D3C6) XP	Cell signaling	4796
EGFR	Cell signaling	2232
ERK1/2 (3A7)	Cell signaling	9107
pERK1/2 (Thr202/Tyr204)	Cell signaling	9101
FGFR1 (D8E4) XP	Cell signaling	9740
GAPDH Mouse	Cell signaling	5174
GAPDH Rabbit	Cell signaling	97166
NEDD4 (C5F5)	Cell signaling	3607
PLCγ1 (M156)	Abcam, Amsterdam, The Netherlands	ab41433
pPLCγ1 (Ser1248)(D25A9)	Cell signaling	8713
SOX2	Abcam	ab97959
SPRY2	Abcam	ab60719
SPRY2	Upstate, Vienna, Austria	07-524
α-Tubulin (DM1A)	Cell signaling	3873
β-Tubulin (9F3)	Cell signaling	2128
Ubiquitin (E4I2J)	Cell signaling	43124

Abbreviations: C-casitas b-lineage lymphoma (c-CBL); epidermal growth factor receptor (EGFR); extracellular signal-regulated kinase (ERK); fibroblast growth factor receptor 1 (FGFR1); glyceraldehyde-3-phosphate dehydrogenase (GAPDH); neural precursor cell-expressed developmentally downregulated protein 4 (NEDD4); phospholipase Cγ1 (PLCγ1); Sprouty2 (SPRY2).

## Data Availability

All obtained datasets supporting the conclusions of this article are included within the article.

## Supplementary Materials

The following supporting information can be downloaded at https://www.mdpi.com/article/10.3390/cells13231967/s1, Figure S1: Direct comparison of endogenous FGFR1 versus overexpression of FGFR1-EGFP in U251 and SF126 cells; Figure S2: Antibody control of the FGFR1 immunoprecipitation and NEDD4 Western blot of anti-FGFR1 immunoprecipitates; Figure S3: Uncropped Western blot images.
