# Supplementary material for: Sprouty2 Regulates Endocytosis and Degradation of Fibroblast Growth Factor Receptor 1 in Glioblastoma Cells
Source: Cells. 2024 Nov 28;13(23):1967. doi: 10.3390/cells13231967 (PMC11639775; doi:10.3390/cells13231967)
Supplement: Supplementary file 1 [file cells-13-01967-s001.zip › Figure S1.pdf]

Figure S1

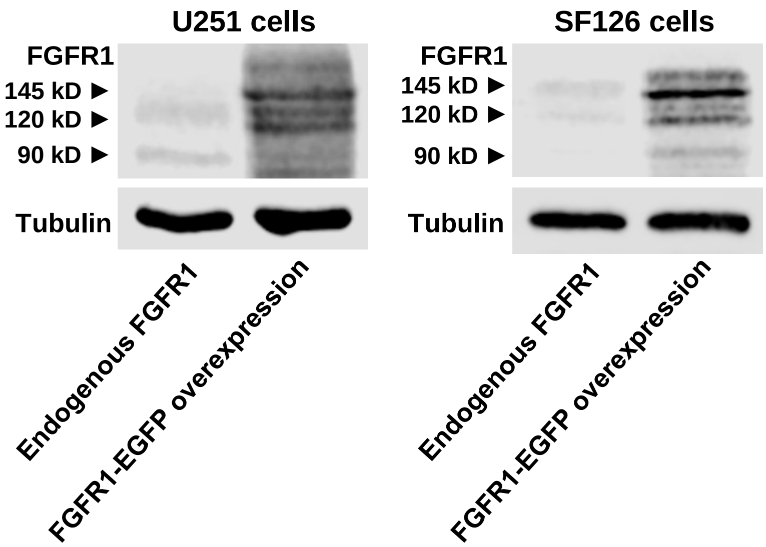

Figure S1. Direct comparison of endogenous FGFR1 versus overexpression of FGFR1-EGFP in U251 and SF126 cells.
