# Supplementary material for: Sprouty2 Regulates Endocytosis and Degradation of Fibroblast Growth Factor Receptor 1 in Glioblastoma Cells
Source: Cells. 2024 Nov 28;13(23):1967. doi: 10.3390/cells13231967 (PMC11639775; doi:10.3390/cells13231967)
Supplement: Supplementary file 1 [file cells-13-01967-s001.zip › Figure S2.pdf]

Figure S2

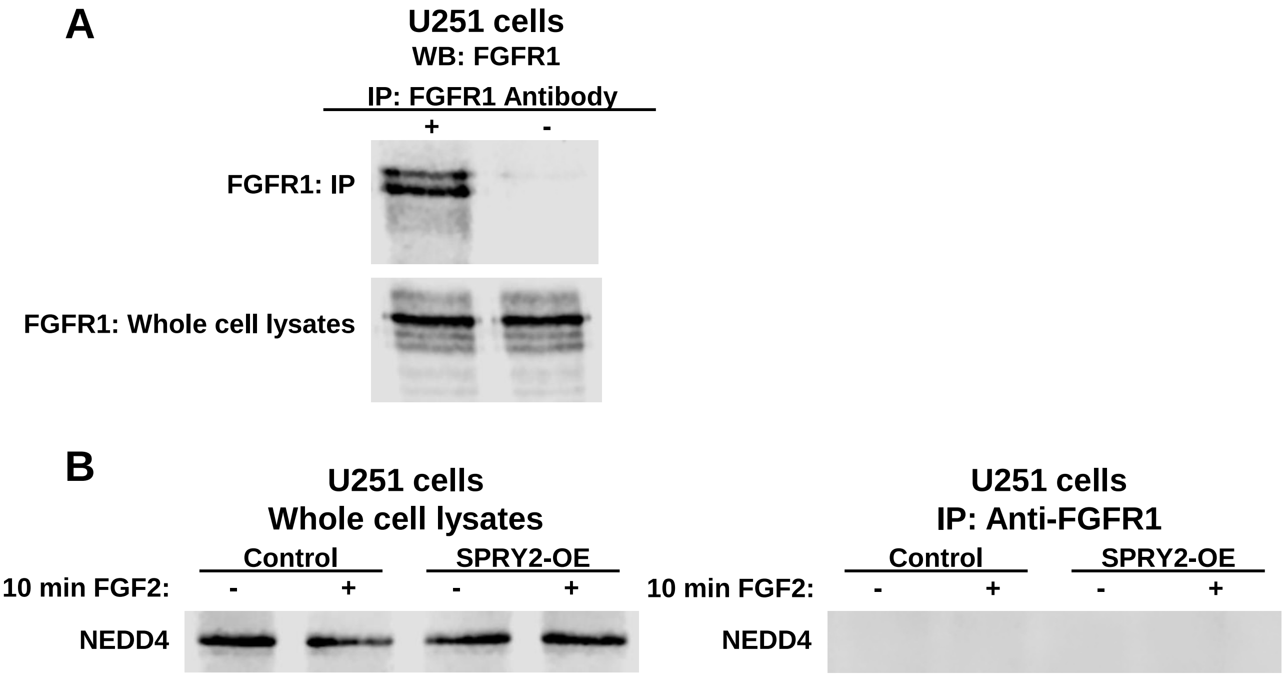

Figure S2. Antibody control of the FGFR1 immunoprecipitation and NEDD4 Western blot of anti-FGFR1 immunoprecipitates. (A) FGFR1 was not detected in lysates incubated with plain beads without antibody incubation. (B) NEDD4 was not detected in anti-FGFR1 immunoprecipitates, although it was present in whole-cell lysates.
