# Supplementary material for: Sprouty2 Regulates Endocytosis and Degradation of Fibroblast Growth Factor Receptor 1 in Glioblastoma Cells
Source: Cells. 2024 Nov 28;13(23):1967. doi: 10.3390/cells13231967 (PMC11639775; doi:10.3390/cells13231967)
Supplement: Supplementary file 1 [file cells-13-01967-s001.zip › Figure S3 uncropped Western blot images.pdf]

Figure S3

Uncropped Western blot images

Figure 1

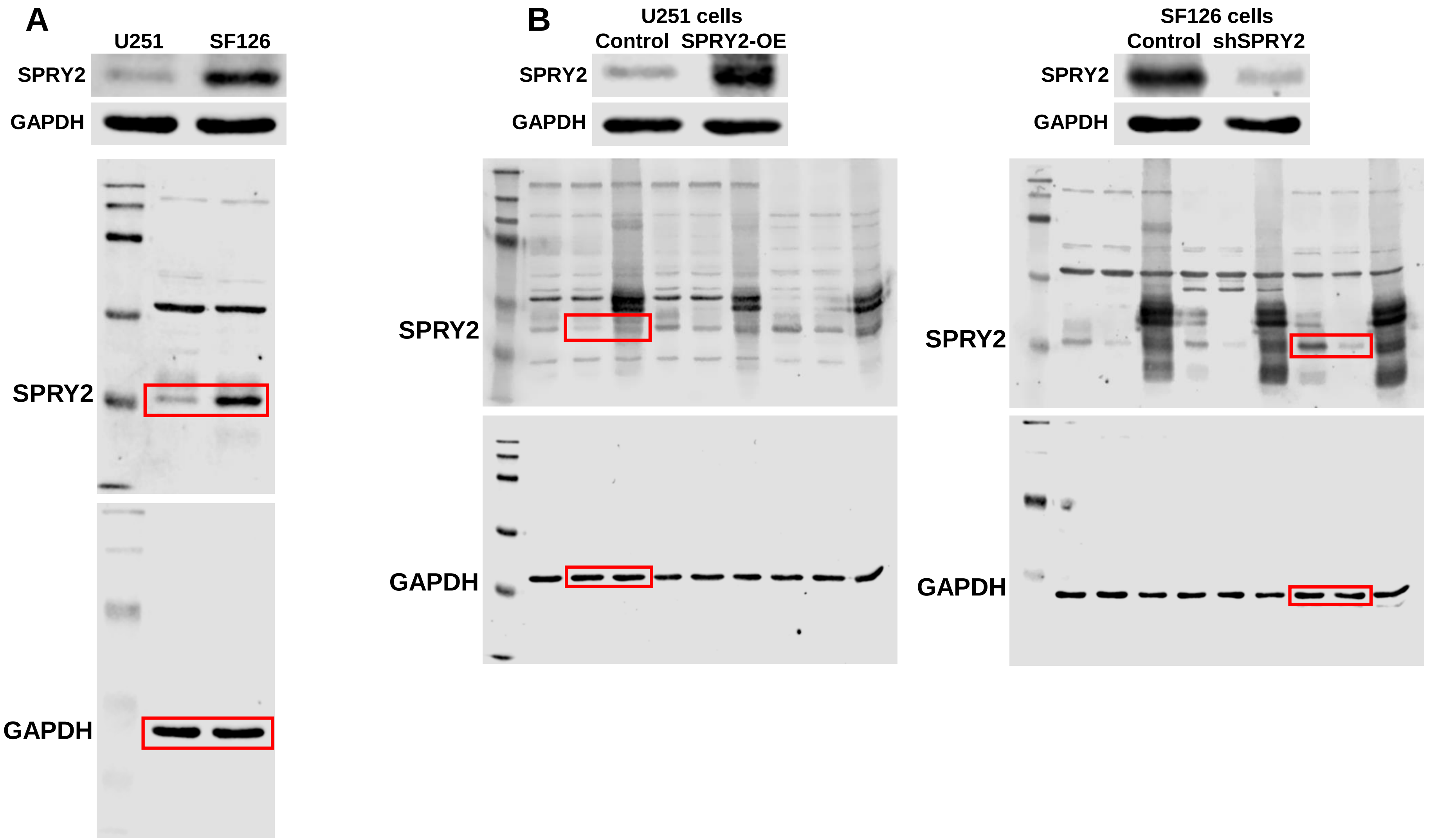
A
U251
SF126
SPRY2
GAPDH
SPRY2
GAPDH
B
U251 cells
Control
SPRY2-OE
SPRY2
GAPDH
SPRY2
GAPDH
SF126 cells
Control
shSPRY2
SPRY2
GAPDH
SPRY2
GAPDH

Figure 5

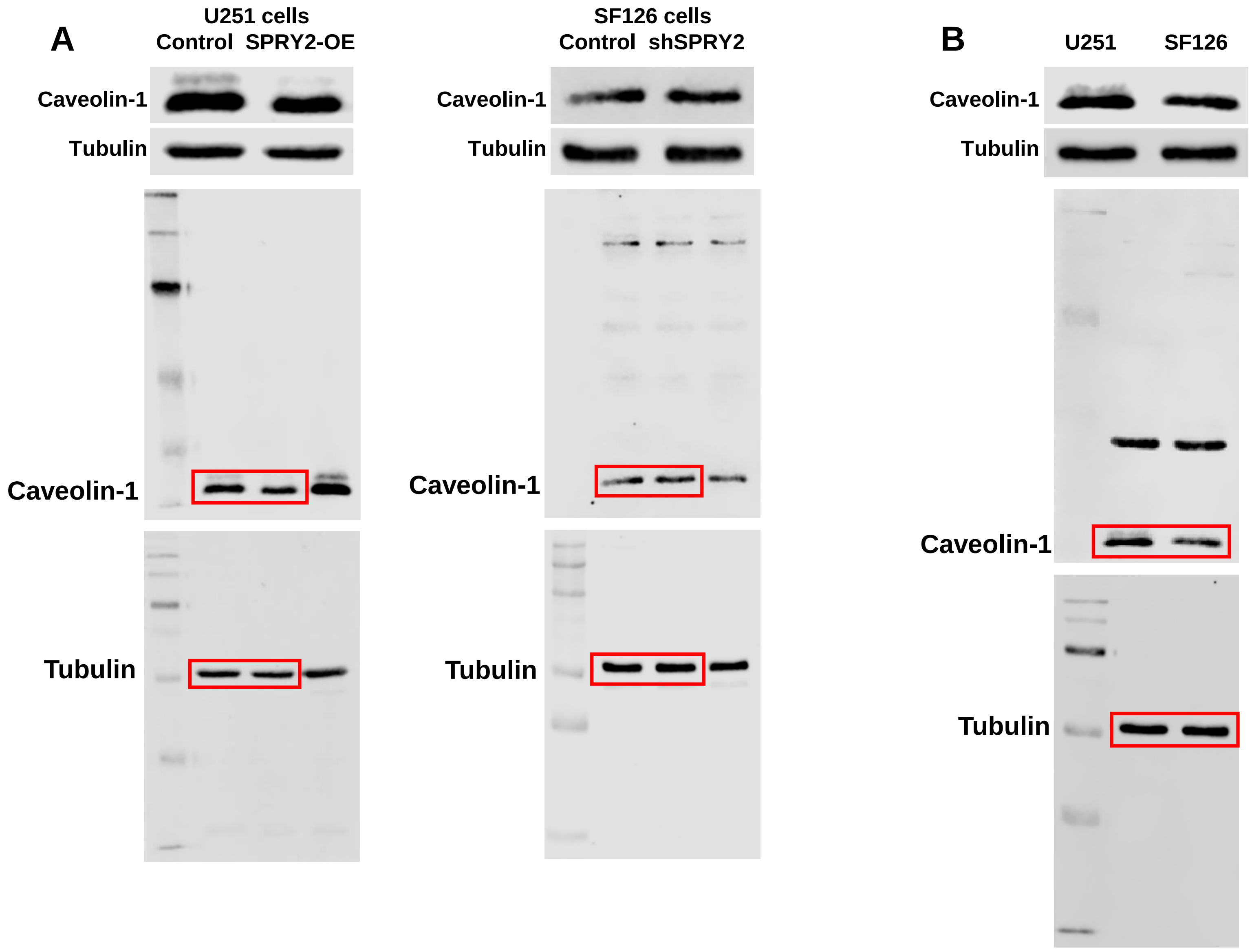

A
U251 cells
Control
SPRY2-OE
Caveolin-1
Tubulin
SF126 cells
Control
shSPRY2
Caveolin-1
Tubulin
B
U251
SF126
Caveolin-1
Tubulin

Figure 7A,C

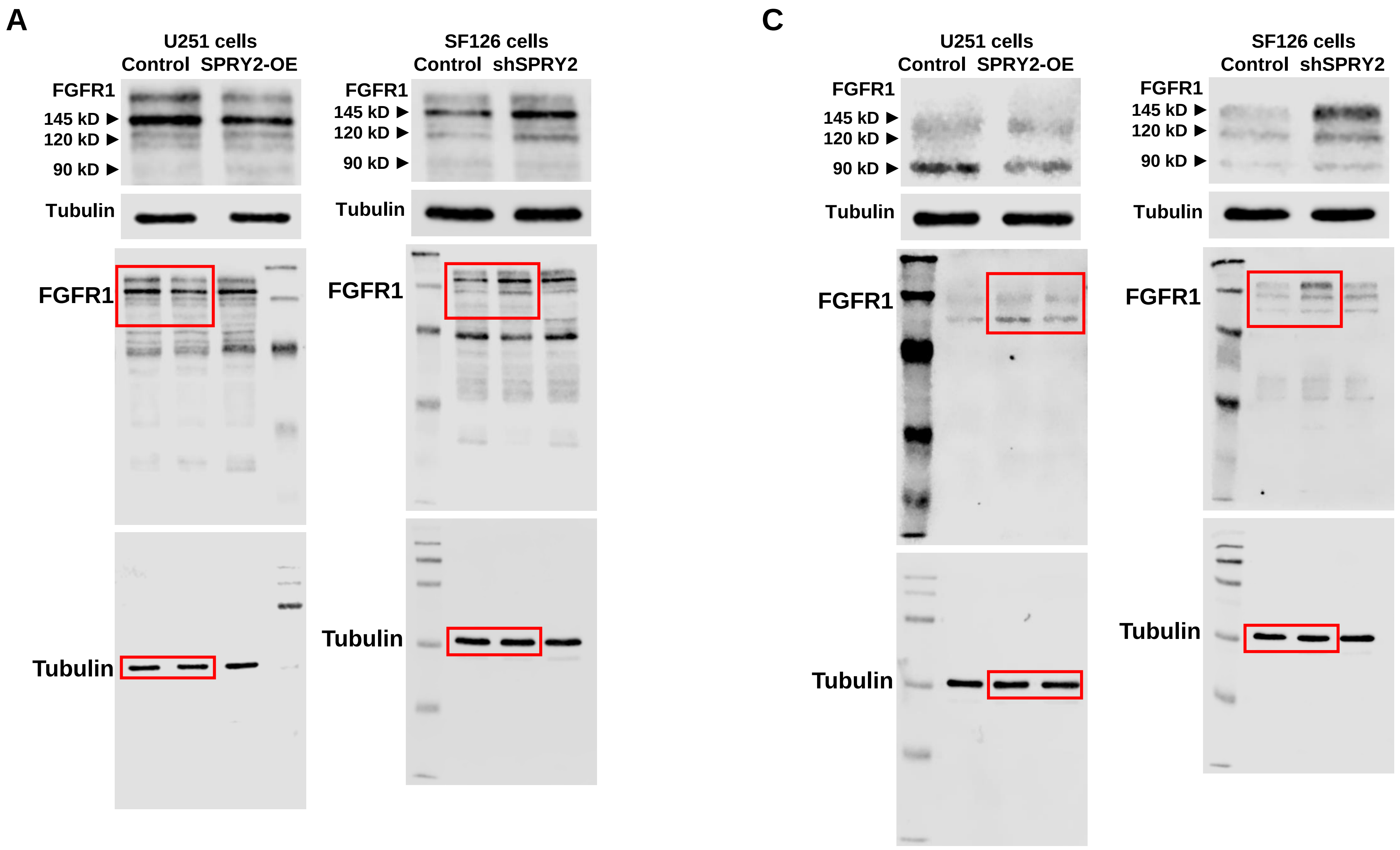
A
U251 cells
Control
SPRY2-OE
FGFR1
145 kD
120 kD
90 kD
Tubulin
SF126 cells
Control
shSPRY2
FGFR1
145 kD
120 kD
90 kD
Tubulin
FGFR1
Tubulin
FGFR1
Tubulin
C
U251 cells
Control
SPRY2-OE
FGFR1
145 kD
120 kD
90 kD
Tubulin
SF126 cells
Control
shSPRY2
FGFR1
145 kD
120 kD
90 kD
Tubulin
FGFR1
Tubulin
FGFR1
Tubulin

Figure 7D

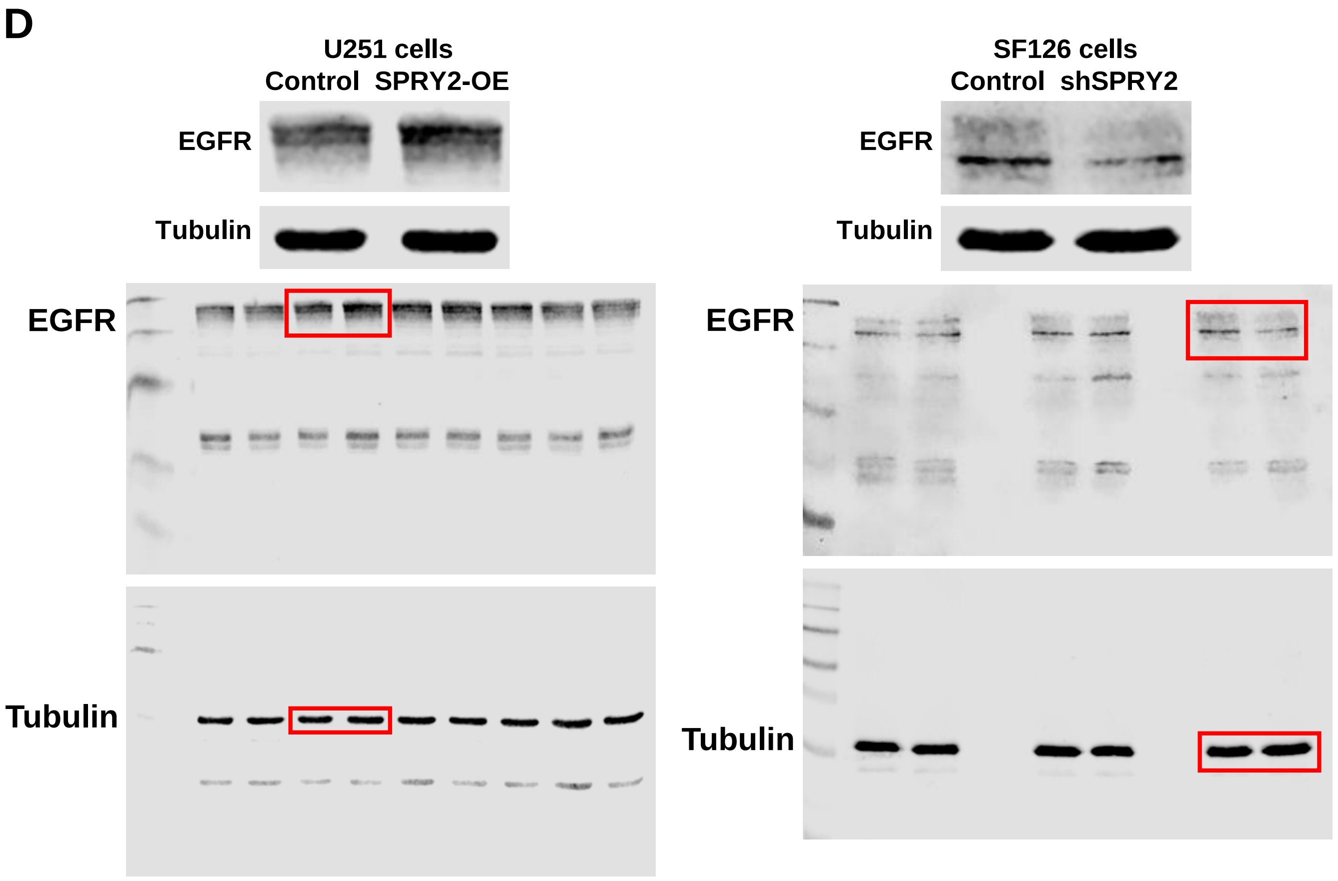
D
U251 cells
Control SPRY2-OE
EGFR
Tubulin
EGFR
Tubulin
SF126 cells
Control shSPRY2
EGFR
Tubulin
EGFR
Tubulin

Figure 8A

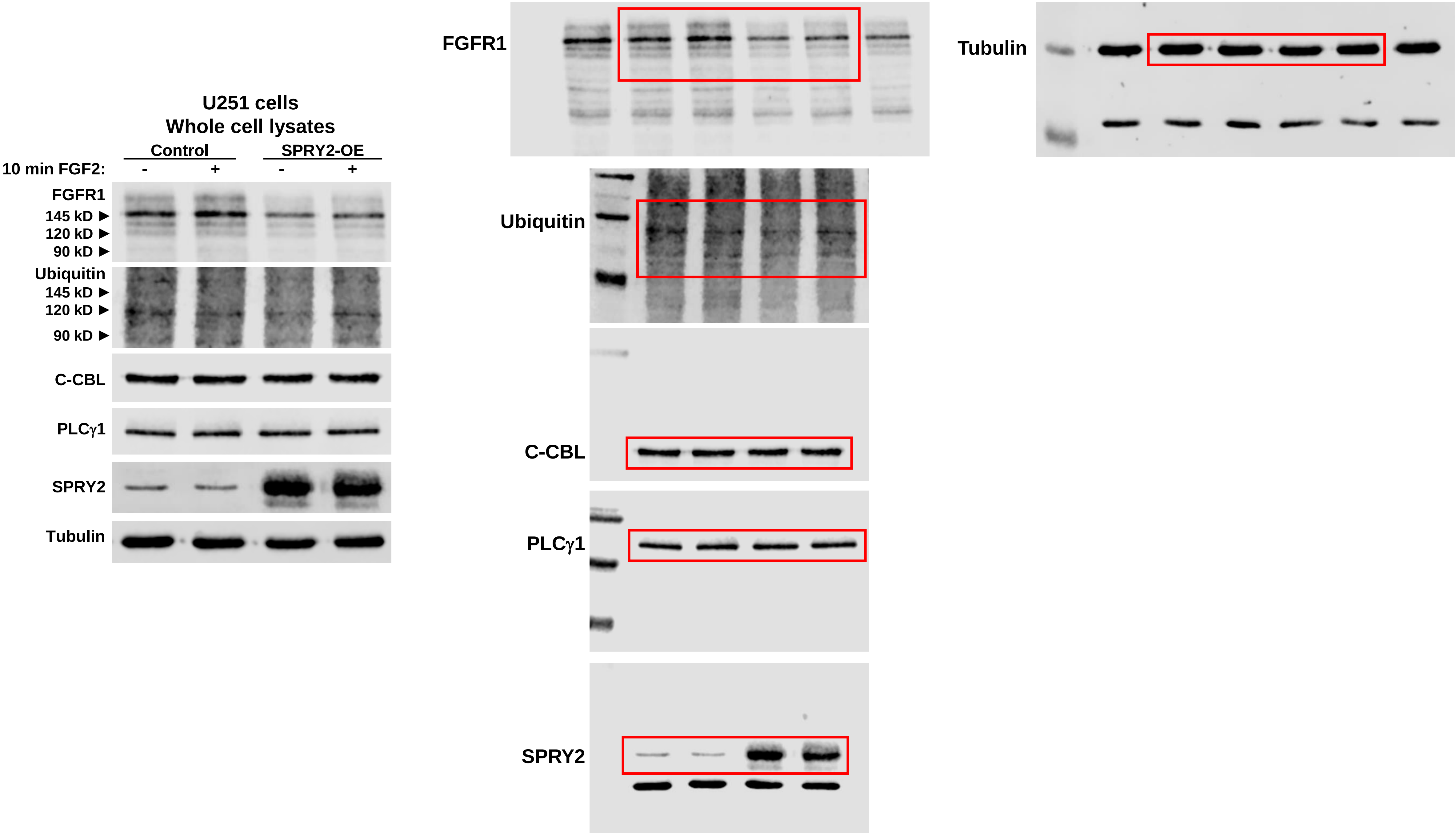
U251 cells
Whole cell lysates
Control
SPRY2-OE
10 min FGF2:
-
+
-
+
FGFR1
145 kD ▶
120 kD ▶
90 kD ▶
Ubiquitin
145 kD ▶
120 kD ▶
90 kD ▶
C-CBL
PLCγ1
SPRY2
Tubulin
FGFR1
Tubulin
Ubiquitin
C-CBL
PLCγ1
SPRY2

Figure 8B

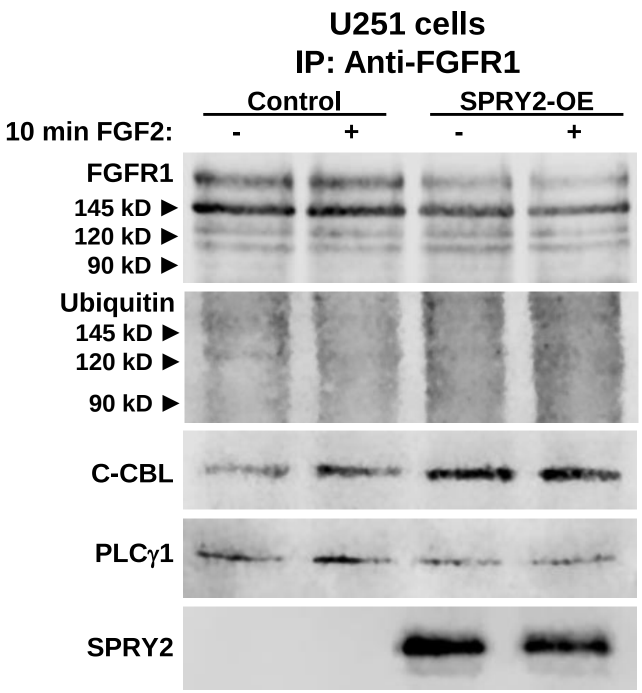

U251 cells
IP: Anti-FGFR1
Control
SPRY2-OE
10 min FGF2:
-
+
-
+
FGFR1
145 kD ▶
120 kD ▶
90 kD ▶
Ubiquitin
145 kD ▶
120 kD ▶
90 kD ▶
C-CBL
PLCγ1
SPRY2

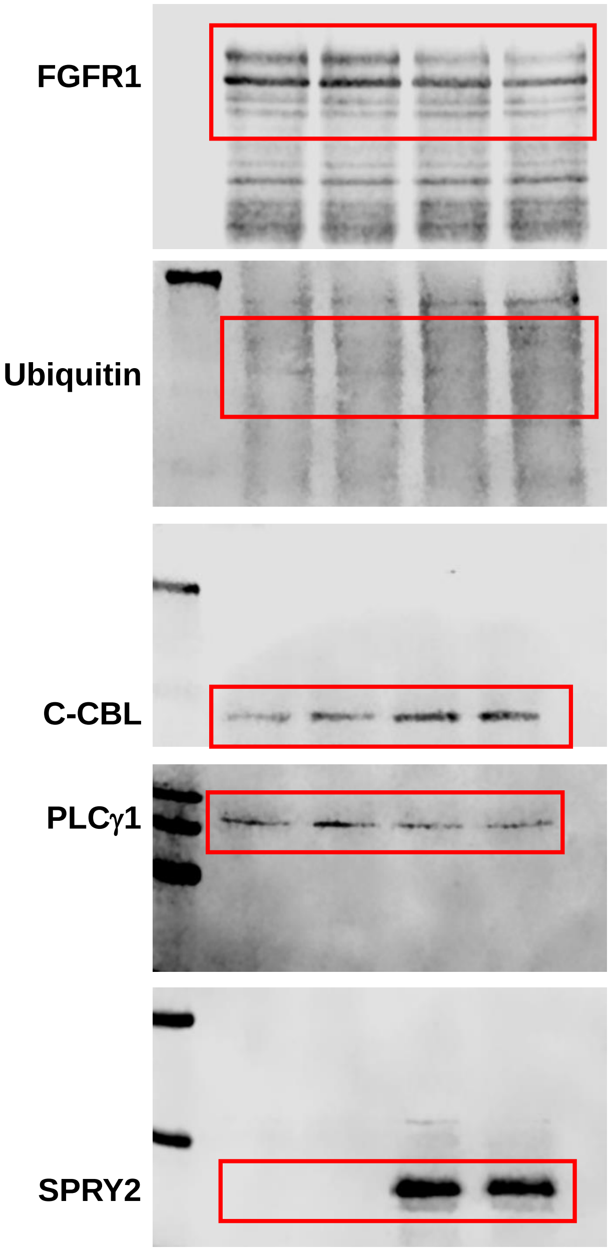

FGFR1
Ubiquitin
C-CBL
PLCγ1
SPRY2

Figure 9

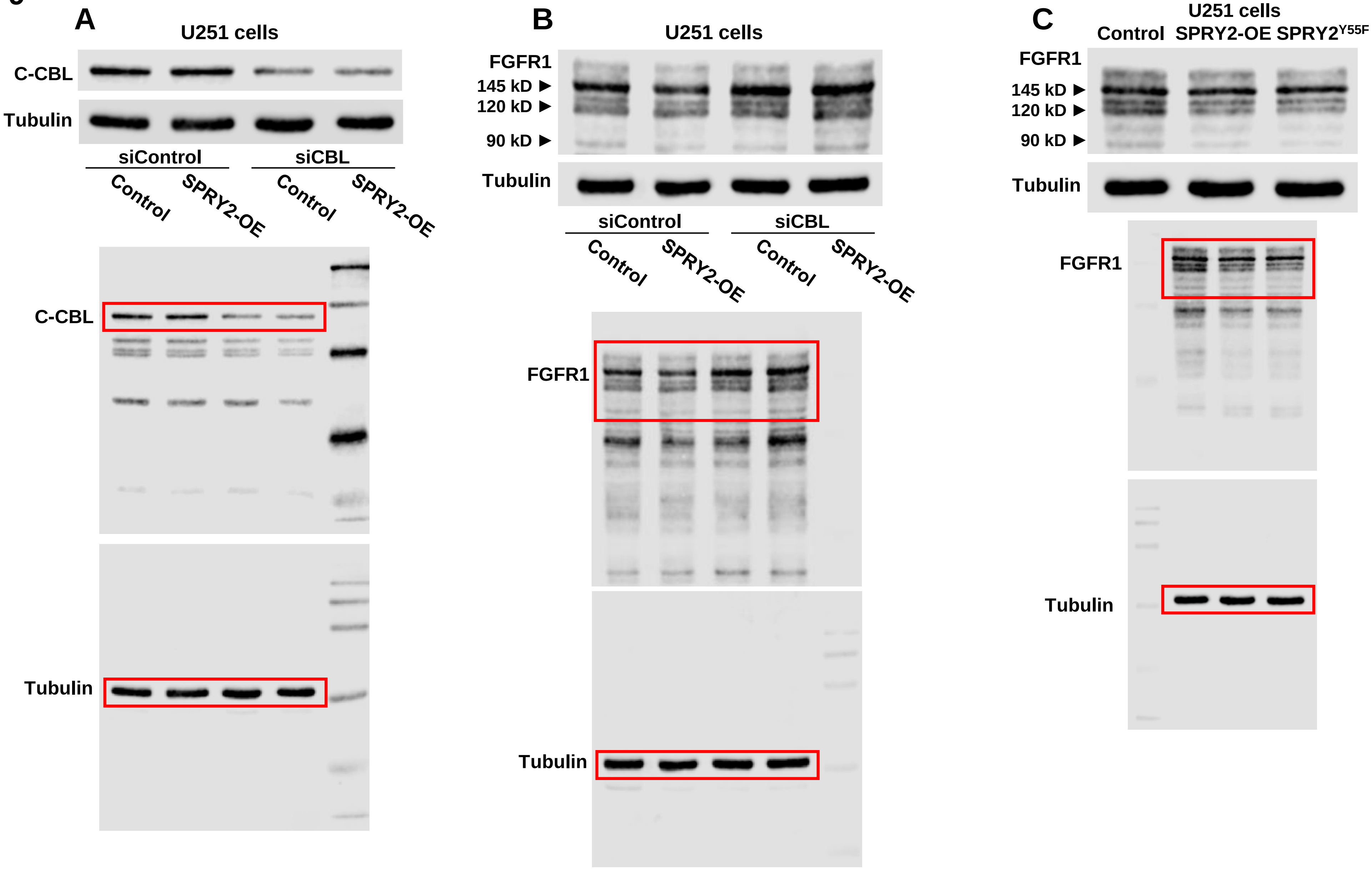
A
U251 cells
C-CBL
Tubulin
siControl
siCBL
Control
SPRY2-OE
Control
SPRY2-OE
C-CBL
Tubulin
B
U251 cells
FGFR1
145 kD
120 kD
90 kD
Tubulin
siControl
siCBL
Control
SPRY2-OE
Control
SPRY2-OE
FGFR1
Tubulin
C
U251 cells
Control
SPRY2-OE
SPRY2Y55F
FGFR1
145 kD
120 kD
90 kD
Tubulin
FGFR1
Tubulin

Figure 10

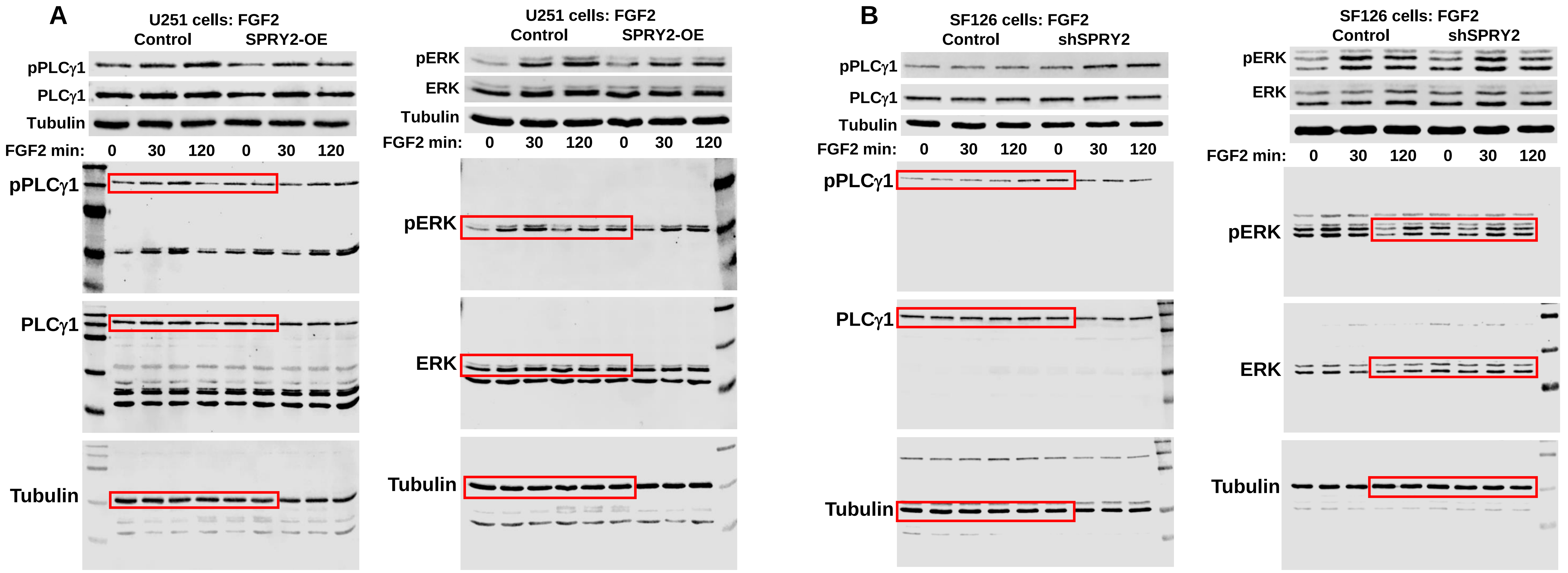

A
U251 cells: FGF2
Control
SPRY2-OE
pPLCγ1
PLCγ1
Tubulin
FGF2 min: 0 30 120 0 30 120
pPLCγ1
PLCγ1
Tubulin
U251 cells: FGF2
Control
SPRY2-OE
pERK
ERK
Tubulin
FGF2 min: 0 30 120 0 30 120
pERK
ERK
Tubulin
B
SF126 cells: FGF2
Control
shSPRY2
pPLCγ1
PLCγ1
Tubulin
FGF2 min: 0 30 120 0 30 120
pPLCγ1
PLCγ1
Tubulin
SF126 cells: FGF2
Control
shSPRY2
pERK
ERK
FGF2 min: 0 30 120 0 30 120
pERK
ERK
Tubulin

Figure 11

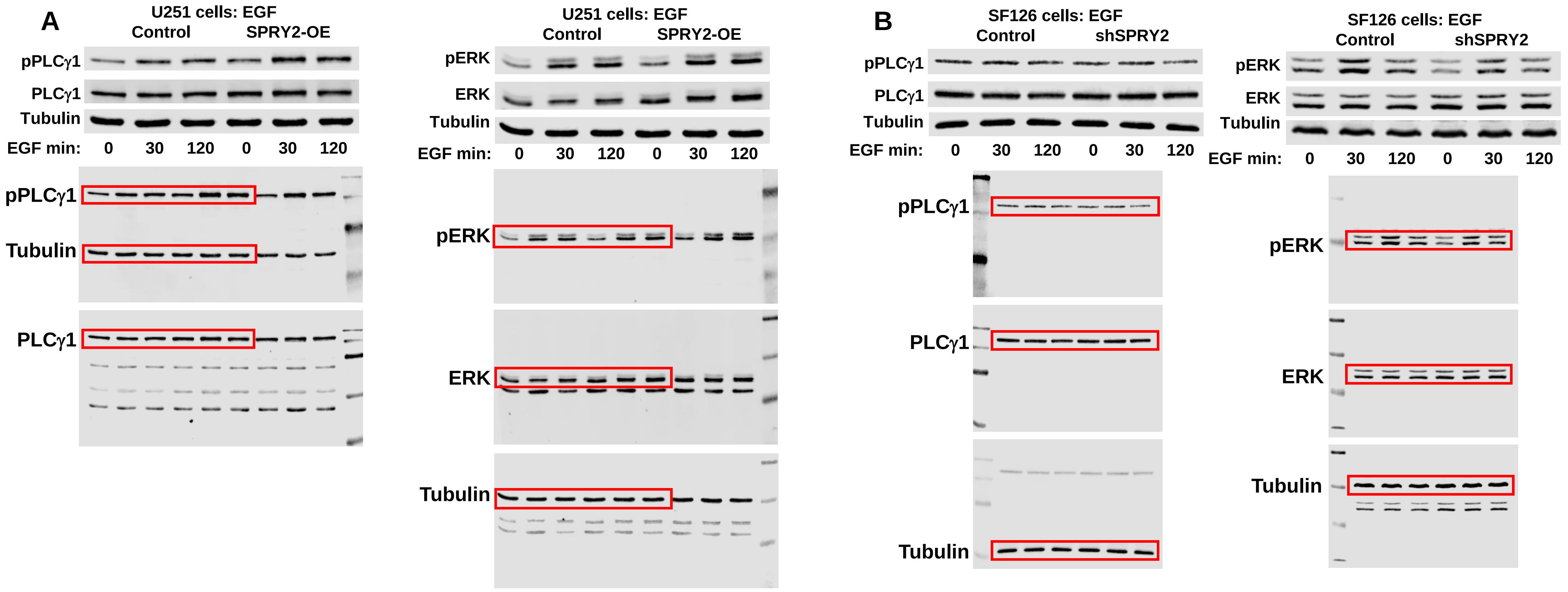
A
U251 cells: EGF
Control
SPRY2-OE
pPLCγ1
PLCγ1
Tubulin
EGF min: 0 30 120 0 30 120
pPLCγ1
Tubulin
PLCγ1
U251 cells: EGF
Control
SPRY2-OE
pERK
ERK
Tubulin
EGF min: 0 30 120 0 30 120
pERK
ERK
Tubulin
B
SF126 cells: EGF
Control
shSPRY2
pPLCγ1
PLCγ1
Tubulin
EGF min: 0 30 120 0 30 120
pPLCγ1
PLCγ1
Tubulin
SF126 cells: EGF
Control
shSPRY2
pERK
ERK
Tubulin
EGF min: 0 30 120 0 30 120
pERK
ERK
Tubulin

Figure 12

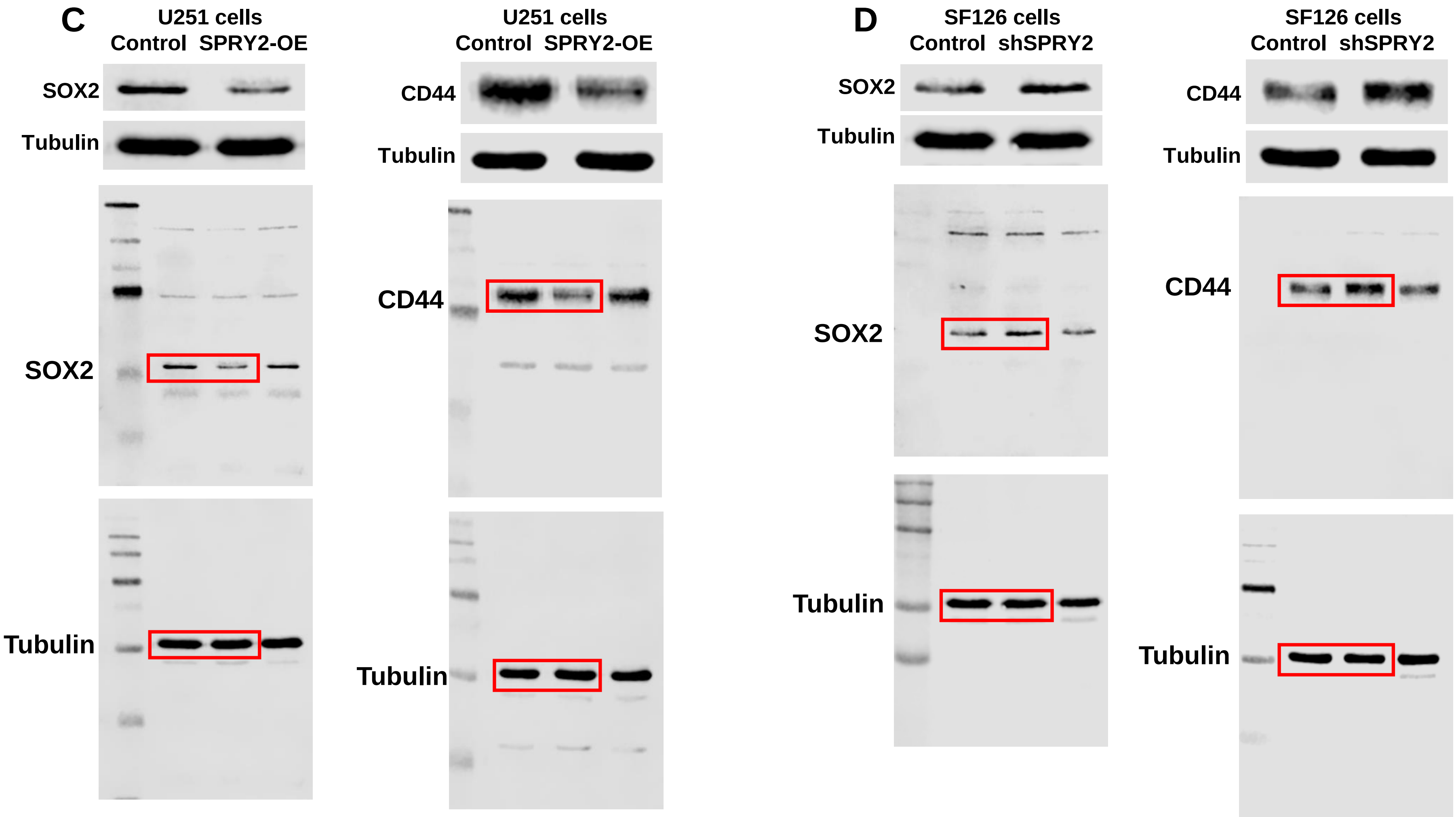

C
U251 cells
Control SPRY2-OE
SOX2
Tubulin
U251 cells
Control SPRY2-OE
CD44
Tubulin
SOX2
Tubulin
CD44
Tubulin
D
SF126 cells
Control shSPRY2
SOX2
Tubulin
SF126 cells
Control shSPRY2
CD44
Tubulin
SOX2
Tubulin
CD44
Tubulin
